# Radiation-induced ferroptosis via liposomal delivery of 7-Dehydrocholesterol

**DOI:** 10.1186/s12951-025-03303-3

**Published:** 2025-03-26

**Authors:** Jianwen Li, Shuyue Zhan, Wei Yang, He Zhang, Xinrui Ma, Fanghui Chen, Amy Li, Pakteema Tong, Fangchao Jiang, Zhengwei Cao, Ian Delahunty, Jiayi Wang, Yufei Wu, Zhi Liu, Zibo Li, Yong Teng, Libin Xu, Jin Xie

**Affiliations:** 1 Department of Chemistry, University of Georgia, Athens, GA 30602 USA; 2 Department of Radiology, University of North Carolina at Chapel Hill, Chapel Hill, NC 27599 USA; 3 Department of Hematology and Medical Oncology & Winship Cancer Institute, Emory University School of Medicine, Atlanta, GA 30322 USA; 4 Department of Medicinal Chemistry, University of Washington, Seattle, WA 98195 USA

**Keywords:** Liposomes, 7-dehydrocholesterol, Radiosensitizer, Lipid peroxidation, Ferroptosis

## Abstract

**Background:**

Ferroptosis is an emerging cell death mechanism characterized by uncontrolled lipid peroxidation. However, selectively inducing ferroptosis in cancer cells remains a challenge.

**Methods:**

We explore an approach that enables ferroptosis induction through external radiation. The key component of this technology is 7-dehydrocholesterol (7DHC), a natural biosynthetic precursor of cholesterol. To facilitate delivery, we demonstrate that 7DHC, like cholesterol, can be incorporated into the lipid layer of liposomes. To enhance targeting, we also introduced NTS_mut_, a ligand for the neurotensin receptor 1 (NTSR1), which is overexpressed in multiple malignancies, into liposomes.

**Results:**

Under radiation, 7DHC reacts with radiation-induced reactive oxygen species (ROS), initiating a radical chain reaction with polyunsaturated fatty acids (PUFAs) in cell membranes. This process results in direct lipid peroxidation and subsequent ferroptotic cell death. In vivo studies demonstrate that NTS_mut_-conjugated, 7DHC-loaded liposomes (N-7DHC-lipos) effectively accumulate in tumors and significantly enhance the efficacy of radiation therapy.

**Conclusion:**

While conventional radiosensitizers primarily target DNA and its repair mechanisms, our study introduces a strategy to enhance radiotherapy by specifically activating ferroptosis within the irradiated area, thereby minimizing systemic toxicity. Such a strategy of controlled activation of ferroptosis offers a favorable therapeutic index and potentially opens avenues for clinical application.

**Supplementary Information:**

The online version contains supplementary material available at 10.1186/s12951-025-03303-3.

## Graphical Abstract

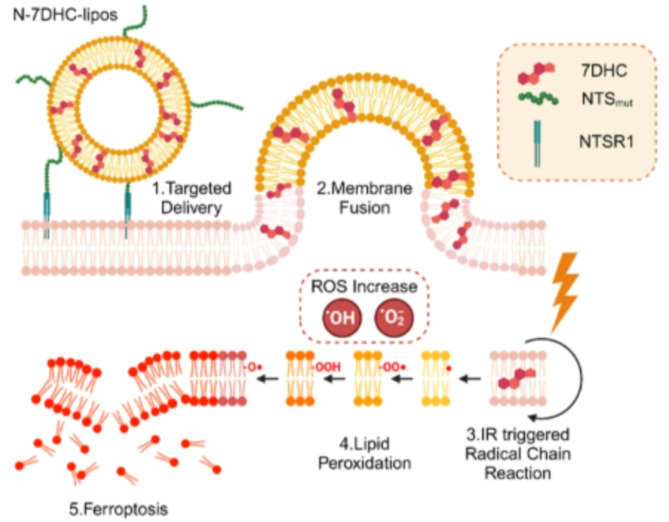

## Background

Ferroptosis is characterized by the failure of glutathione-dependent antioxidant defenses, leading to uncontrolled lipid peroxidation and eventual cell death [1]. Ferroptosis is distinct from apoptosis and necrosis and, as an alternative cell death mechanism, has the potential to overcome resistance associated with conventional chemotherapy and radiotherapy [2–4]. This is supported by the observation that many cancers show increased susceptibility to ferroptosis due to their aberrant regulation of critical ferroptosis checkpoints such as the xCT/GSH/GPX4 axis, TGF-ZEB1, and PML/PGC1α [5–10]. In addition, non-mutational states associated with a mesenchymal-like phenotype and resistance to standard radiotherapy also show sensitivity to ferroptosis [10, 11].

Current ferroptosis inducers, including erastin, RSL3, and FIN56, primarily target xCT and GPX4 [12]. However, many of these compounds are highly hydrophobic, making them unsuitable for direct systemic administration. Efforts have been made to develop nanoparticle carriers capable of delivering these inducers to tumor sites [13–17]. Despite the progress, challenges remain regarding targeting specificity and the risk of collateral damage to healthy tissues. An ideal strategy would involve delivering ferroptosis inducers as prodrugs that can be activated in situ by internal or external stimuli, thus maximizing targeting specificity [18]. However, progress in this area has been limited.

Herein we investigate 7DHC, a zoosterol and a biosynthetic precursor of cholesterol [19], as a novel ferroptosis inducer that can be activated by irradiation. With a diene in the B-ring, 7DHC is extremely susceptible to radical attack, forming a pentadienyl radical [20]. The resultant sterol radical and the corresponding peroxyl radical can then react with another 7DHC or a nearby PUFA, initiating radical chain reactions between lipid molecules [19]. We postulate that such a unique property of 7DHC can be exploited to enable radiation-induced ferroptosis. Meanwhile, 7DHC outside the radiation field is expected to gradually metabolized to cholesterol, which is safely absorbed by the host.

As a cholesterol analog, 7DHC has poor water solubility and cannot be administered directly. To overcome this problem, we explore liposomes as carriers for 7DHC, inspired by the structural similarity between 7DHC and cholesterol and the ability of the latter to be embedded in phospholipid layers. For tumor targeting, we imparted NTS_mut_, a ligand with strong binding affinity to neurotensin receptor 1 (NTSR1) [21, 22], onto the nanoparticles. NTSR1 is upregulated in multiple types of cancer, including non-small cell lung cancer (NSCLC) [23–26], but not in normal tissues [23]. Moreover, NTSR1 is recognized as a negative prognostic marker for lung cancer patients [23], making it an appealing and druggable target. In this study, we evaluated the efficacy of our 7DHC liposomes both in in vitro and in vivo using lung cancer cells and tumor models.

## Results and discussion

### Synthesis and characterization of 7DHC-lipos

**Fig. 1:**
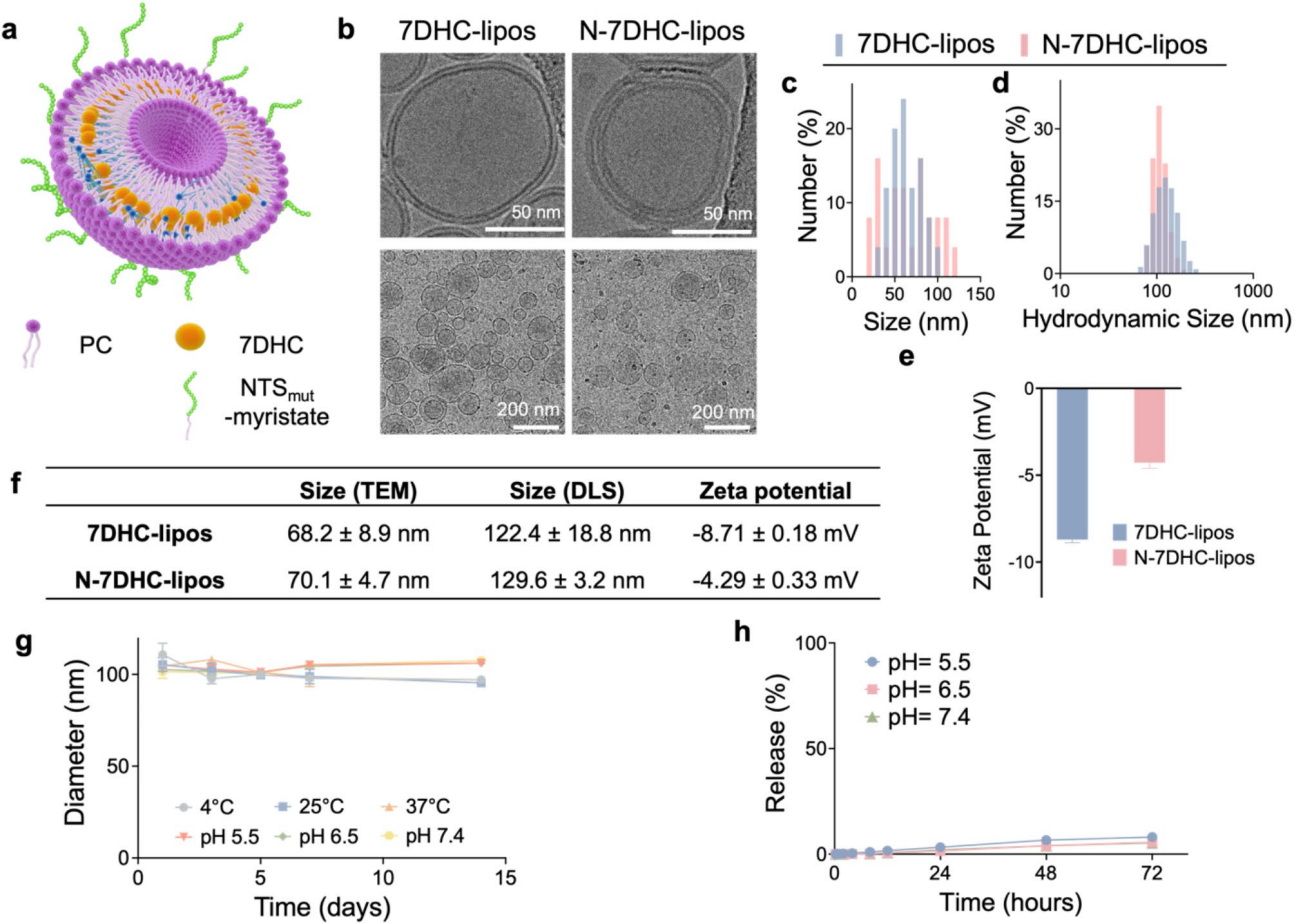
Physiochemical characterization of the nanoparticles. (**a**) Schematic illustration of the composition of N-7DHC-lipos. PC, phosphatidylcholine. (**b**) Cryo-TEM images of 7DHC-lipos and N-7DHC-lipos. Scale bars, 50 μm (upper) and 200 μm (lower). (**c**) Size distribution of 7DHC-lipos and N-7DHC-lipos, based on cyro-TEM results and analysis of more than 50 nanoparticles by Image J. (**d**) Hydrodynamic sizes of 7DHC-lipos and N-7DHC-lipos, measured by DLS in 1×PBS. (**e**) Zeta potential of 7DHC-lipos and N-7DHC-lipos. (**f**) Table summering sizes and zeta potentials of 7DHC-lipos and N-7DHC-lipos. (**g**) Hydrodynamic size change of 7DHC-lipos under different temperatures (4 °C, 25 °C, 37 °C) and pH values (pH 5.5, pH 6.5, pH 7.4) over two weeks incubation in 1×PBS. (**h**) 7DHC release from 7DHC-lipos, measured in buffer solutions with pH 7.4, 6.5, and 5.5 at 37 °C (*n* = 3 independent experiments). Data are presented as mean ± SD

We synthesized 7DHC-loaded liposomes (7DHC-lipos) by the conventional thin-film hydration method [27]. Briefly, we dissolved phosphatidylcholine and 7DHC at a molar ratio of 3:1 in chloroform and removed the solvent by evaporation (Fig. 1a). We then resuspended the liposomes in Tris buffer (pH = 8.0) and subjected the solution to extrusion through a 100-nm filter to obtain 7DHC-lipos. The 7DHC loading capacity (drug loaded relative to total weight, LC%) and encapsulation efficiency (drug encapsulated relative to initial drug added, EE%) were 10.4% and 63.7%, respectively.

Cryogenic transmission electron microscopy (cryo-TEM) revealed that 7DHC-lipos have a bilayer structure with a size of 74.3 ± 6.5 nm (Fig. 1b&c). Dynamic light scattering (DLS) found an average hydrodynamic diameter of 105.7 nm (Fig. 1d). The surface of the nanoparticles carries a slight negative charge in PBS (-8.71 mV, Fig. 1e, f). It is worth noting that the loading capacity, nanoparticle size, and surface properties of 7DHC-liposomes are comparable to liposomes encapsulating cholesterol instead of 7DHC (Fig. S1a&b). Furthermore, 7DHC-liposomes are stable in solutions under different temperatures and pH values and show negligible size changes after more than two weeks of incubation (Fig. 1g). Like cholesterol, 7DHC exhibits minimal release from the liposomes in neutral or acidic conditions (Fig. 1h & Fig. S1c).

### N-7DHC-lipos and their uptake by Cancer cells

For tumor targeting, we introduced NTS_mut_ into 7DHC-lipos by including myristoylated NTS_mut_ during liposome synthesis. NTS_mut_ (Lys-Pro-(NMe-Arg)-Arg-Pro-Tyr-Tle-Leu) is a neurotensin analog that has shown comparable avidity to NTSR1 to neurotensin but much greater stability [28, 29]. The EE% of myristoylated NTS_mut_, based on LC-MS, was 32.6%, and the molar ratio between NTS_mut_ and phosphatidylcholine in the resulting NTS_mut_-conjugated 7DHC-lipos (referred to as N-7DHC-lipos for simplicity) was 1:37. N-7DHC-lipos are similar in size to unmodified 7DHC-lipos, as shown by cryo-TEM and DLS (Fig. 1b-d), and their zeta potential was − 4.29 mV (Fig. 1e&f).

**Fig. 2:**
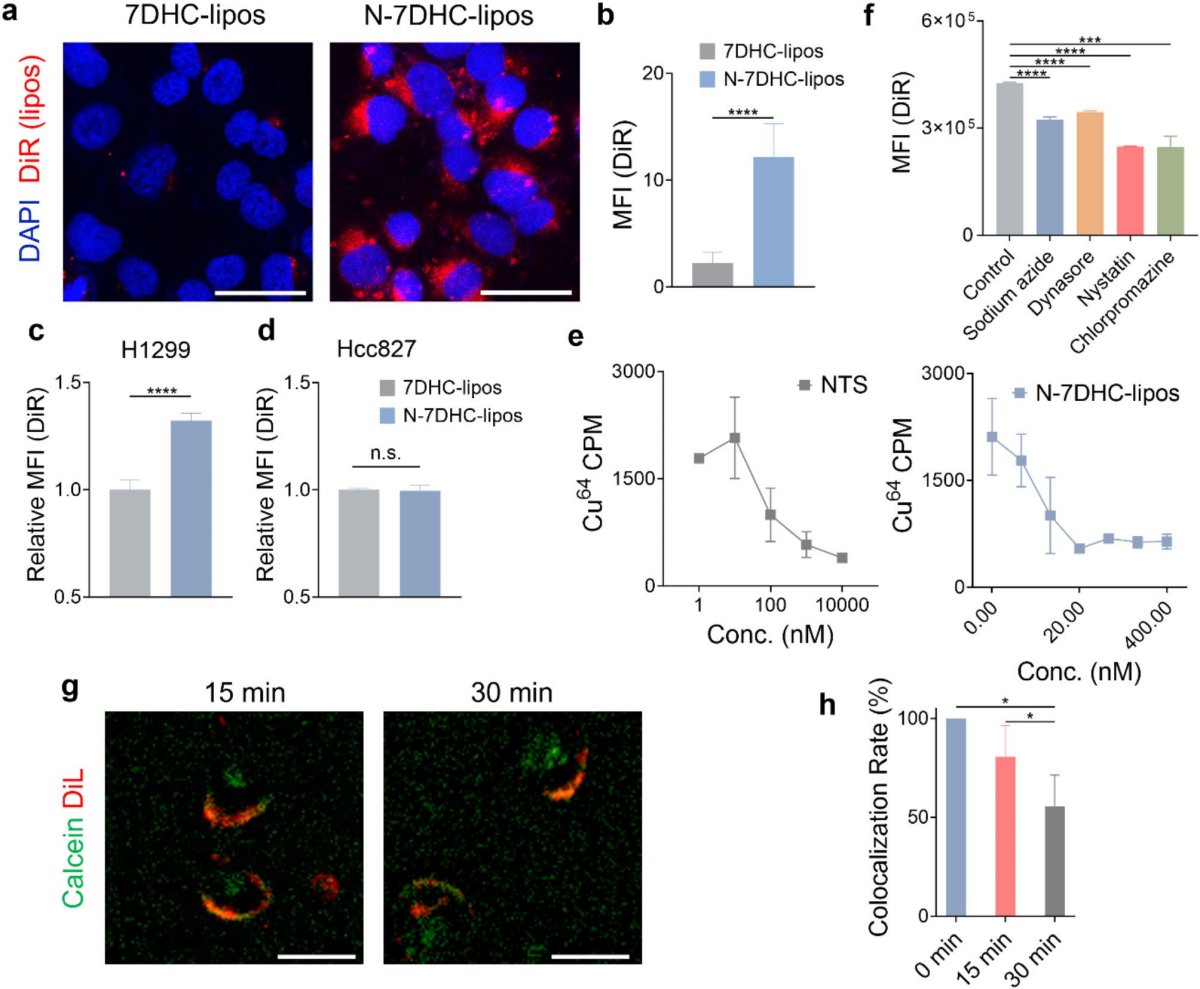
Cellular uptake of N-7DHC-lipos. (**a**) Fluorescence microscopy comparing uptake of N-7DHC-lipos and 7DHC-lipos by H1299 cells at 4 h. Scale bars, 50 μm. (**b**) Mean fluorescence intensity (MFI) of DiR in cells, analyzed by Image J based on microscopy results of (**a**). (**c**,** d**) Flow cytometry comparing uptake of N-7DHC-lipos and 7DHC-lipos by NTSR1-positive H1299 cells (**c**) and NTSR1-negative Hcc827 cells (**d**) (*n* = 3 biologically independent samples). (**e**) Competitive binding curve of N-7DHC-lipos (right) and neurotensin (NTS) (left) (*n* = 3 biologically independent samples). (**f**) Inhibition of DiR-labeled N-7DHC-lipos uptake by endocytosis inhibitors, evaluated with H1299 cells using flow cytometry (*n* = 3 biologically independent samples). (**g**) Fluorescence microscopy analysis of N-7DHC-lipos fusion with cell membranes, tested with live H1299 cells. N-7DHC-liposomes were loaded with DiL, embedded within the lipid layers, and calcein, encapsulated inside the nanoparticles. An increase in intracellular calcein fluorescence and a decrease in colocalization of the two dyes indicate fusion of N-7DHC-lipos to cell membranes and release of the encapsulated contents into the cytosol. Scale bars, 50 μm. (**h**) Statistical analysis of the change in colocalization between DiL and calcein, based on the microscopy results from (**g**) and analyzed by Image J. The experiment was repeated three times with similar results. Data are presented as mean ± SD. Statistical difference was evaluated using two-tailed Student’s t-test in (b-d), and one-way ANOVA in (f, h). *p < 0.05, ***p < 0.001, ****p < 0.0001; ns, *p* > 0.05

We then examined the cellular uptake of N-7DHC-lipos. We labeled N-7DHC-lipos or 7DHC-lipos (i.e. not coupled with NTS_mut_) with DiR and incubated them with H1299 cells, an NTSR1-positive NSCLC cell line [30]. Fluorescence microscopy revealed significantly increased uptake of N-7DHC-lipos compared to 7DHC-lipos (Fig. 2a&b). Flow cytometry also confirmed a significant difference in uptake between N-7DHC-lipos and 7DHC-lipos (Fig. 2c). In contrast, when tested with NTSR1-negative Hcc827 cells, N-7DHC-lipos exhibited low internalization and showed no significant difference in uptake compared to 7DHC-lipos (Fig. 2d). To validate the NTSR1 targeting, we also performed binding affinity assay using ^64^Cu-labeled neurotensin (^64^Cu-NT-NOTA) as a competitive ligand. While binding of ^64^Cu-NT-NOTA to H1299 cells was effectively inhibited by both N-7DHC-lipos and free NTS_mut_ (Fig. 2e), N-7DHC-lipos showed higher displacement efficiency on a per NTS basis. This higher binding affinity with N-7DHC-lipos is attributed to the multivalency effect, i.e. the advantage of having multiple ligands on a single nanoparticle to allow for simultaneous interactions with more than one target receptors [31].

After binding to NTSR1, N-7DHC-lipos enter cells *via* receptor-mediated endocytosis. This is supported by a significant reduction in uptake when N-7DHC-lipos were co-incubated with sodium azide, a general endocytosis inhibitor (Fig. 2f). To further investigate the internalization mechanism, we also co-incubated N-7DHC-lipos with dynasore and chlorpromazine, which inhibit dynamin GTPase activity and clathrin-coated pit formation [32], respectively. Both agents significantly reduced the uptake of N-7DHC-lipos, indicating that dynamin- and clathrin-dependent endocytosis are involved in particle internalization. In addition, nystatin, an inhibitor of lipid rafts, also inhibited nanoparticle uptake, suggesting that caveolae endocytosis plays a role as well. Of note, both clathrin- and caveolae-mediated endocytosis have been observed by others with liposomes [33, 34]. In contrast, EIPA (5-(N-Ethyl-N-isopropyl)amiloride), a macropinocytosis inhibitor, had no impact on the uptake of N-7DHC-lipos (Fig. S2).

Interestingly, not all 7DHC-lipos were endocytosed by NTSR1-positive cells, as indicated by the observation that none of the endocytosis inhibitors were able to completely block the uptake of N-7DHC-lipos by H1299 cells (Fig. 2f). Instead, a significant number of the nanoparticles fuse directly into the target cell membrane. This was demonstrated by microscopy with N-7DHC-lipos co-loaded with two dyes, calcein and DiL. Calcein is a hydrophilic dye that is encapsulated in the interior of the liposomes, while DiL is a hydrophobic dye that is inserted into the lipid layer of liposomes [35]. Live cell microscopy revealed a significant reduction in the co-localization rate between these the two dyes shortly after nanoparticle incubation with cells (Fig. 2g&h). Specifically, while DiL signals were found almost exclusively on the cell membrane, calcein signals were found largely inside the cells, indicating liposome fusion with the cell membranes and release of the inner payload into the cells. Unlike physiological endocytosis, which is energy dependent and takes hours to occur, the membrane diffusion is a rapid physical process, as evidenced by a ~ 50% reduction in dye colocalization within 30 min of incubation (Fig. 2h). Similarly, when N-7DHC-lipos were dually labeled with hydrophobic NBD-PE and hydrophilic Texas-Red-PE, which form a fluorescence resonance energy transfer (FRET) pair, we observed a decrease in NBD-PE fluorescence intensity with longer incubation in cells, suggesting dye separation due to membrane fusion (Fig. S3). This membrane diffusion capability is advantageous for 7DHC-mediated radiosensitization as the target is unsaturated lipid molecules on cell membranes rather than intracellular molecules.

### Impact of N-7DHC-lipos plus irradiation on cell redox homeostasis

**Fig. 3:**
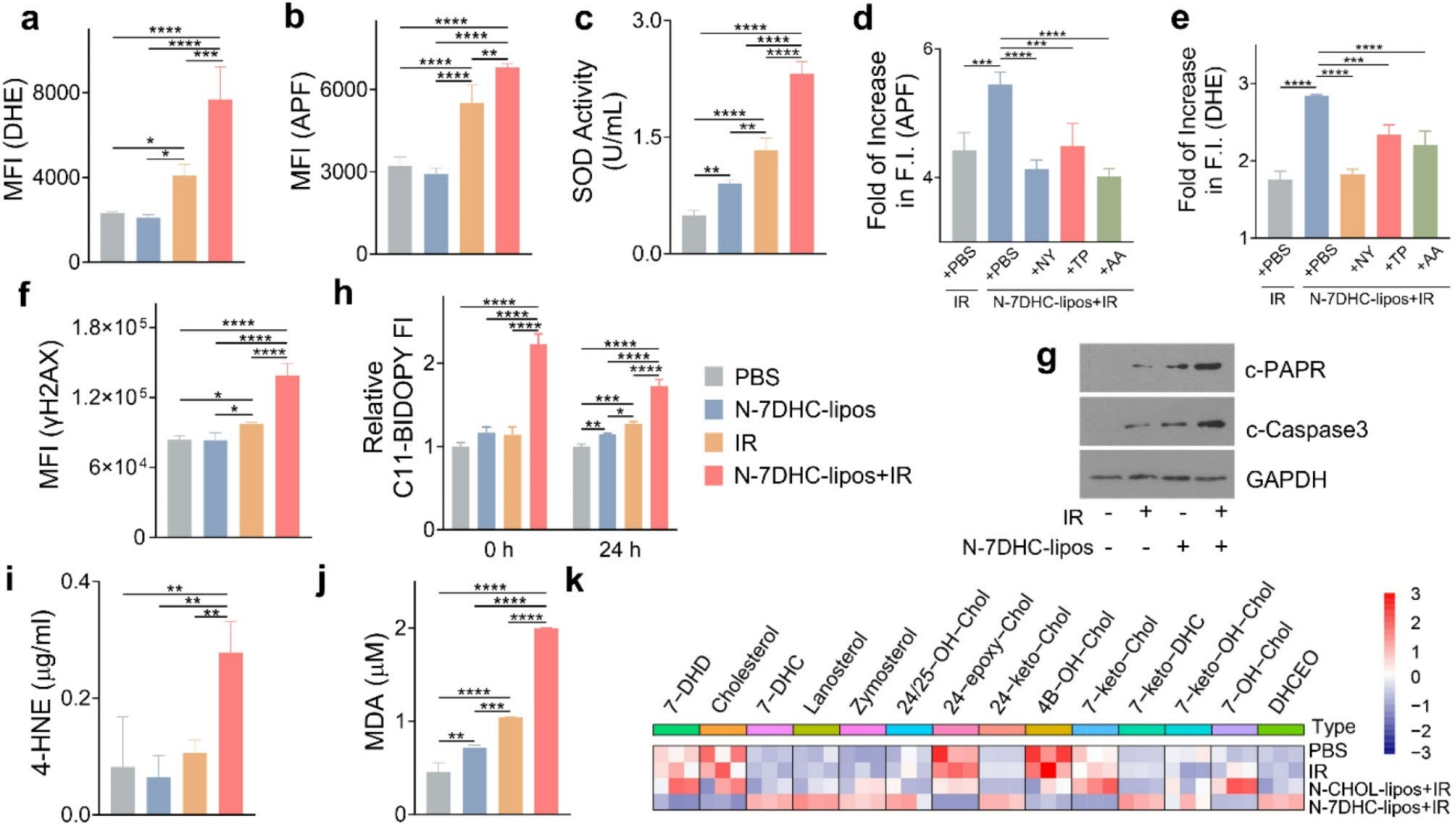
N-7DHC-lipos plus irradiation induces lipid peroxidation, tested with H1299 cells. N-7DHC-lipos were applied at a dose of 50 µg/mL (an equivalent 7DHC dose of 5 µg/mL), and irradiation (IR) was applied at 5 Gy (*n* = 3 biologically independent samples). (**a**,** b**) Influence on cellular superoxide (**a**) and hydroxyl radical (**b**) levels, measured by DHE and APF, respectively. (**c**) Influence on cellular SOD activity. (**d**,** e**) Cellular superoxide (**d**) and hydroxyl radicals (**e**) upon treatment with N-7DHC-lipos plus irradiation while co-incubated with nystatin (NY), tocopherol (TP), or ascorbic acid (AA), measured by DHE and APF, respectively. (**f**) Double-strand breaks, measured by flow cytometry with anti-γH2AX-stained cells. (**g**) Induction of apoptosis, measured by Western blotting analyzing the levels of c-PAPR and c-Caspase3. (**h-j**) Influence on lipid peroxidation, measured by C11-BIDOPY (**h**), 4-HNE (**i**), and TBARS (**j**) assays. (**k**) Intercellular sterol and oxysterol levels, measured by UPLC-MS/MS (*n* = 3 biologically independent samples). Data in the heat map are z-scored. Data are presented as mean ± SD. Statistical difference was evaluated using one-way ANOVA in (a-f, and h-j). *p < 0.05, **p < 0.01, ***p < 0.001, ****p < 0.0001

Next, we investigated the effect of combining N-7DHC-lipos with irradiation on cell oxidative stress. We examined the levels of superoxide and hydroxyl radicals using DHE and APF, respectively, as the corresponding indicators. While N-7DHC-lipos alone had no significant effect on radical levels, they significantly increased both superoxide and hydroxyl radical levels under irradiation (Fig. 3a&b). Consistent with this observation, there was a significant increase in SOD activity in H1299 cells treated with N-7DHC-lipos plus irradiation compared to irradiation alone (Fig. 3c), which is attributed to a cell survival response to the elevated ROS levels. For comparison, we also synthesized cholesterol-encapsulated counterparts, where 7DHC was replaced by cholesterol, termed N-CHOL-lipos. N-CHOL-lipos have similar size and surface properties to N-7DHC-lipos (Fig. S1). Unlike N-7DHC-lipos, however, N-CHOL-lipos showed an insignificant impact on cellular ROS levels (Fig. S4).

The increase in radical levels was effectively suppressed by tocopherol and ascorbic acid, which are lipophilic and hydrophilic radical scavengers, respectively (Fig. 3d&e). Nystatin, an inhibitor of lipid rafts, also attenuated the N-7DHC-lipos-mediated increase in radical levels. These results suggest an interaction between ROS in the cytosol and radical reactions between lipid molecules that drive up cellular oxidative stress. The increase in ROS spread damage to intracellular biomolecules, including DNA, which is evidenced by a significant increase in positive staining for γH2AX (Fig. 3f). This is followed by the induction of apoptosis, as evidenced by marked cleavage of PAPR and caspase 3 (Fig. 3g). In comparison, N-CHOL-lipos plus irradiation had a much less pronounced influence on DNA damage (Fig. S4).

The more profound impact of N-7DHC-lipos mediated radiosensitization, however, was on lipid peroxidation. Specifically, C11-BIDOPY staining detected no enhancement of lipid peroxidation with neither irradiation (5 Gy) nor N-7DHC-lipos (Fig. 3h). On the contrary, shortly after treatment with a combination of N-7DHC-lipos and irradiation, there was a significantly increased level of lipid peroxidation (Fig. 3h), which is attributed to radical chain reactions among unsaturated lipid molecules.

To validate treatment induced lipid peroxidation, we also performed 4-hydroxynonenal (4-HNE) and TBARS assays, which measure by-products from peroxidation of PUFAs. Consistent with the C11-BIDOPY result, irradiation or N-7DHC-lipos alone had an insignificant effect on the level of 4-HNE, whereas the combination of the two caused a significant increase in this peroxidation by-product (Fig. 3i). Similarly, a significant increase in malondialdehyde (MDA) levels was observed with N-7DHC-lipos plus irradiation compared to standalone N-7DHC-lipos or irradiation (Fig. 3j). Taken together, our results suggest that N-7DHC-lipos can effectively enhance irradiation-induced peroxidation among PUFAs.

The radical chain reactions may cause autooxidation among 7DHC molecules, producing oxysterols [20]. To investigate, we treated H1299 cells with N-7DHC-lipos plus irradiation (5 Gy) and harvested the cells for analysis of cellular sterol and oxysterol levels by LC-MS/MS (Fig. 3k). We found that the combination treatment resulted in a significant increase in oxysterols, including DHCEO and 7-keto-DHC (proposed mechanisms in Fig. S5). In particular, the level of 7-keto-DHC, which is a 7DHC-derived oxysterol [36, 37], increased by 33-fold. Note that DHCEO is a biomarker for Smith-Lemli-Opitz Syndrome, a genetic disorder characterized by a deficiency of 7-dehydrocholesterol reductase or DHCR7 (an enzyme that is essential for the conversion of 7DHC to cholesterol), and therefore an imbalance between 7DHC and cholesterol in the affected patients [38–40]. In comparison, N-CHOL-lipos plus irradiation had a much less pronounced enhancement effect on 7-keto-DHC, and an insignificant effect on DHCEO. It was observed that cells treated with N-7DHC-lipos had significantly increased cellular levels of lanosterol and zymosterol (Fig. 3k). This is probably because lanosterol and zymosterol are biosynthetic precursors of 7DHC [41], and their conversion is inhibited by 7DHC.

### N-7DHC-lipos plus irradiation induces ferroptosis

**Fig. 4:**
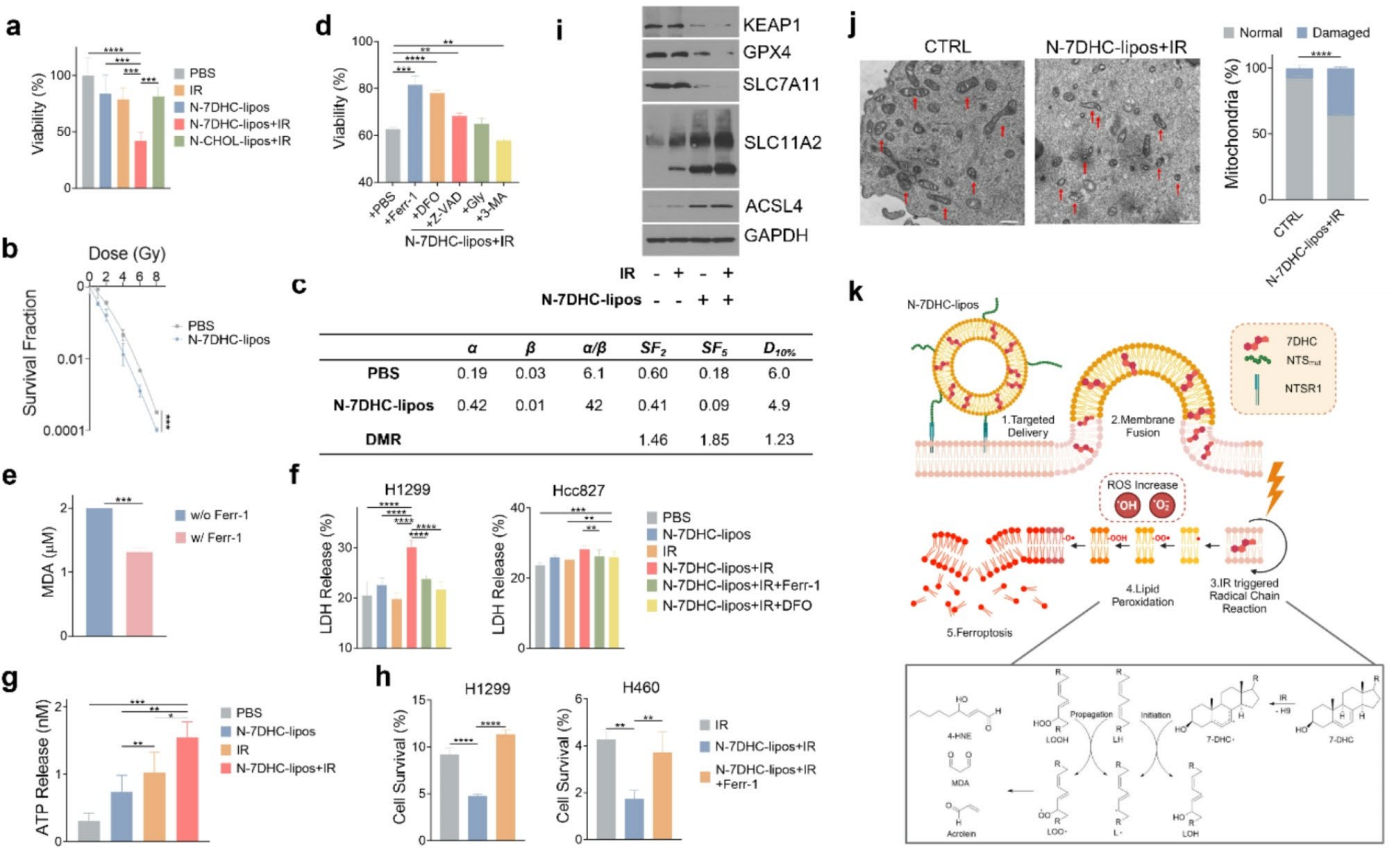
N-7DHC-lipos plus irradiation induces ferroptosis. N-7DHC-lipos were applied at a dose of 50 µg/mL. Except for clonogenicity studies, where a range of radiation doses were tested, irradiation (IR) was applied at 5 Gy. (**a**) Effect of the combination on cell viability, tested in H1299 cells using MTT assays (*n* = 5 biologically independent samples). N-CHOL-lipos plus IR was also tested for comparison. (**b**) Effect on clonogenicity, evaluated by clonogenic assay in H1299 cells. A range of radiation doses (0–10 Gy) was applied, together with N-7DHC-lipos or carrier only. Survival fractions relative to the unirradiated control were fitted to the linear-quadratic model (*n* = 3 biologically independent samples). (**c**) Summary of data fitting, based results from (**b**). *α* and *β*, fitting coefficients. *SF*_*2*_ and *SF*_*5*_, survival fractions at 2 and 5 Gy, respectively. *D*_*10%*_, dose required to reduce the survival fraction to 10%. DMR, dose modifying ratio, based on *SF*_*2*,_*SF*_*5, and*_*D*_*10%.*_ (**d**) Viability of H1299 cells that were treated with N-7DHC-lipos plus IR, and co-incubated with cell death inhibitors, including Ferr-1, DFO, Z-VAD-FMK (Z-VAD), glycine (Gly), and 3-methyladenine (3-MA) (*n* = 5 biologically independent samples). (**e**) Levels of lipid peroxidation when H1299 cells were treated with N-7DHC-lipos and IR while co-incubated with or without Ferr-1, measured by TBARS assay (*n* = 3 biologically independent samples). (**f**) LDH release, measured in H1299 and Hcc827 cells (*n* = 5 biologically independent samples). Cells were treated with N-7DHC-lipos plus IR, while co-incubating with Ferr-1 or DFO. (**g**) ATP release from cells treated with N-7DHC-lipos plus IR, tested in H1299 cells (*n* = 5 biologically independent samples). (**h**) Clonogenic assay, tested in both H1299 and H460 cells. Cells were treated with N-7DHC-lipos plus IR, with or without co-incubation with Ferr-1 (*n* = 3 biologically independent samples). (**i**) Western blot analysis of ferroptosis-related markers, including KEAP1, GPX4, SLC7A11, SCL11A2, and ACSL4, in H1299 cells treated with N-7DHC-lipos and IR, alone or in combination. PBS, IR alone, and N-7DHC-lipos were tested for comparison. (**j**) (Left) TEM images of H1299 cells treated with N-7DHC-lipos plus IR. Red arrows point to mitochondria. Scale bars, 500 nm (upper) and 200 nm (lower). (Right) Bar graphs showing the percentage of normal and damaged mitochondria, based on TEM results. Compared to the untreated control (CTRL), many more damaged mitochondria were observed in cells treated with the combination of N-7DHC-lipos and IR, as manifested by shrunken size and condensed structure. (**k**) Schematic illustration of ferroptosis induced by N-7DHC-lipos plus IR. Data are presented as mean ± SD. Statistical difference was evaluated using one-way ANOVA in (a, d, and f-h), and two-tailed Student’s t-test in (b, e, and j). *p < 0.05, **p < 0.01, ***p < 0.001, ****p < 0.0001

Next, we evaluated the ability of N-7DHC-lipos to enhance radiation-induced cell death in H1299 cells. N-7DHC-lipos (50 µg/mL) (Fig. S6) and irradiation (5 Gy) alone (Fig. 4a) had limited effect on viability. In contrast, their combination resulted in a marked decrease in cell survival (Fig. 4a). Clonogenicity also revealed radiosensitizing effects of N-7DHC-lipos, manifested by a significant reduction in cell colonies compared to radiation alone over a range of radiation doses (0–8 Gy) (Fig. 4b). We fit the survival fraction data to the Liner-Quadratic model. There is a marked increase in α from 0.19 to 0.42 (Fig. 4b&c), echoing observations by others that radiosensitizers predominantly causes α-sensitization [42]. The dose modifying factors (DMFs) based on the survival fraction data at 2 and 5 Gy were 1.46 and 1.85, respectively, suggesting that N-7DHC-lipos is as an effective radiosensitizer. Note that N-7DHC-lipos alone cause minimal toxicity to cells in the absence of irradiation (Fig. 4a).

We then investigated the mechanism underlying the radiosensitization. As mentioned above, N-7DHC-lipos are capable to inducing apoptosis under irradiation (Fig. 3g). However, Z-VAD-FMK, a pan-caspase inhibitor, only moderately attenuated the cell death induced by N-7DHC-lipos in combination with irradiation. Glycine and 3-methyladenine, inhibitors of necrosis and autophagy, respectively, also failed to rescue cells treated with this combination. Conversely, ferrostatin-1 (Ferr-1) and deferoxamine (DFO), two inhibitors of ferroptosis, effectively alleviated the viability decline (Fig. 4d). These results suggest that ferroptosis, which is characteristic of excessive lipid peroxidation, is a primary mechanism driving cell death in response to the combination. This is supported by the observation that Ferr-1 significantly reduced the level of lipid peroxidation in cells treated with N-7DHC-lipos plus irradiation (Fig. 4e).

Furthermore, N-7DHC-lipos plus irradiation resulted in a significant increase in lactate dehydrogenase (LDH) release from H1299, indicating cell membrane disruption as a consequence of lipid peroxidation (Fig. 4f). The membrane disruption was further supported by the increased adenosine triphosphate (ATP) release in cells treated with N-7DHC-lipos and irradiation (Fig. 4g). Meanwhile, co-treatment with Ferr-1 or DFO almost completely reversed the increase in LDH release. Consistent with these findings, we found that Ferr-1 can effectively alleviate the reduction of clonogenicity in H1299 and H460 cells (Fig. 4h). Notably, no LDH release was observed in NTSR1-negative Hcc827 cells (Fig. 4f).

Furthermore, Western blotting showed that cystine/glutamate transporter (SLC7A11) and GPX4, which are involved in the canonical defense against ferroptosis, were downregulated in cells treated with N-7DHC-lipos plus irradiation (Fig. 4i). Kelch-like ECH-associated protein 1 (KEAP1), which recent studies suggest regulates ferroptosis in a SLC7A11/GPX4-independent manner [43], was also downregulated. These data further confirm the induction of ferroptosis by the combination. Interestingly, SLC11A2 (solute carrier family 11 member 2, also known as DMT1), which transports iron into the cytosol, as well as long-chain acyl-CoA synthetase 4 (ACSL4), which incorporates PUFAs such as arachidonic acid into phospholipids, were also elevated in cells treated with N-7DHC-lipos plus irradiation. The influx of iron promotes Fenton reaction that in turn enhances lipid peroxidation, and the incorporation of PUFAs make the lipid membrane more susceptible to radical chain reactions triggered by 7DHC. Both effects sensitize cancer cells to ferroptosis.

Last but not least, we performed TEM on cells treated with 7DHC-lipos plus irradiation (Fig. 4j). We observed typical features of ferroptosis, including mitochondrial shrinkage, increase in membrane density, and disappearance of cristae [44–46]. Taken together, our studies suggest that N-7DHC-lipos under irradiation caused ferroptosis of cancer cells (Fig. 4k).

### Tumor targeting and safety of N-7DHC-lipos

**Fig. 5:**
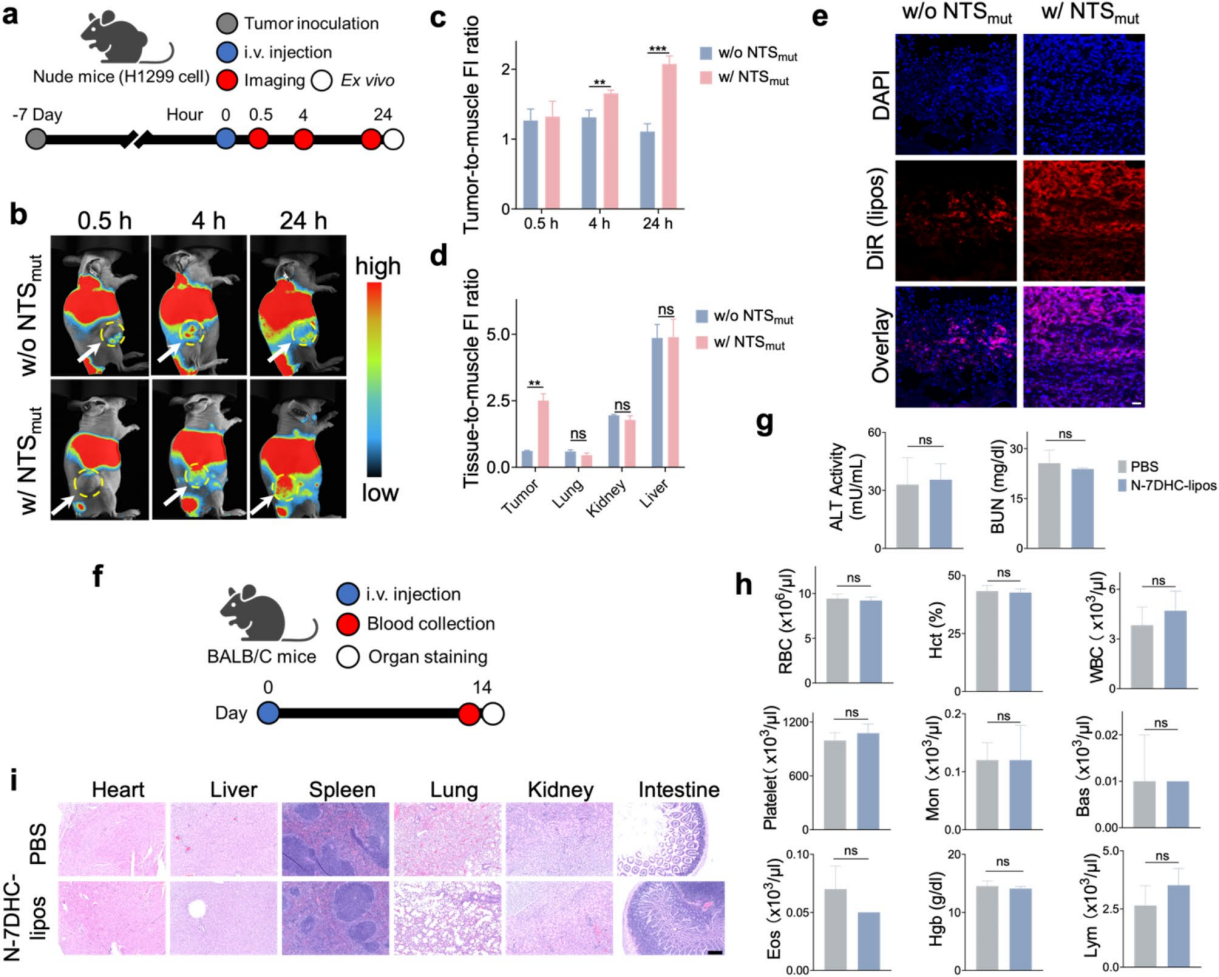
Biodistribution and safety of N-7DHC-lipos. (**a**) Scheme of the biodistribution experiments, which were performed in H1299 tumor-bearing nude mice. 7DHC-lipos, labeled with DiR, and conjugated with NTS_mut_ (w/ NTS_mut_) or without NTS_mut_ (w/o NTS_mut_) were administered i.v. Whole-body fluorescence imaging was performed 0.5, 4, and 24 h after nanoparticle injection (*n* = 3 mice). (**b**) Representative whole-body fluorescence images. (**c**) Tumor-to-muscle fluorescence ratios, based on ROI analysis of imaging results from (**b**). (**d**) Uptake of 7DHC-lipos, w/ and w/o coupling with NTS_mut_, based on ROI analysis of ex vivo imaging results with dissected organs, which was normalized to signals in the muscle. (**e**) Fluorescence microscopy of tumor tissues taken from both w/ NTS_mut_ and w/o NTS_mut_ groups. Blue, DAPI; red, 7DHC-lipos. Scale bar, 50 μm. (**f**) Scheme of the toxicity study. N-7DHC-lipos in PBS (10 mg/kg) or PBS alone were injected i.v. to healthy BALB/C mice (*n* = 3 mice). The animals were euthanized after two weeks. Blood and major organs were collected for CBC, biochemistry, and histopathology. (**g**) ALT and BUN results. (**h**) Selected CBC results. RBC, red blood cells; Hct, hematocrit test; WBC, white blood cells; Hgb, hemoglobin; Lym, lymphocytes; Mon, monocytes; Eos, eosinophils; Bas, basophils. (**i**) H&E staining of major organ tissues. Scale bar, 200 μm. Data are presented as mean ± SD. Statistical difference was evaluated using two-tailed Student’s t-test in (c-d and g-h). **p < 0.01, ***p < 0.001; ns, *p* > 0.05

We then evaluated the tumor targeting of N-7DHC-lipos in vivo. Briefly, we labeled the N-7DHC-lipos with DiR and administered the nanoparticles intravenously (i.v., 10 mg/kg) into nude mice bearing H1299 tumors (*n* = 3 mice). DiR-labeled 7DHC-lipos were injected for comparison. We acquired whole-body fluorescence images at 0.5, 4, and 24 h using an animal fluorescence scanner (Fig. 5a). Compared to 7DHC-lipos, N-7DHC-lipos show significantly increased tumor accumulation at 24 h (Fig. 5b). Based on region of interest (ROI) analysis, the tumor-to-muscle ratio was increased 2.08-fold with N-7DHC-lipos, compared to 1.11-fold with 7DHC-lipos (Fig. 5c).

After the 24-hour scan, the animals were euthanized, and major organs were harvested. Consistent with the in vivo results, ex vivo imaging showed increased tumor accumulation with N-7DHC-lipos compared to 7DHC-lipos (Fig. 5d). Microscopic imaging also indicates more favorable tumor uptake with N-7DHC-lipos than with 7DHC-lipos (Fig. 5e). Meanwhile, no difference in uptake was observed between N-7DHC-lipos and 7DHC-lipos in major organs including the lung, liver, and kidney (Fig. 5d).

We next evaluated the toxicity of N-7DHC-lipos. Briefly, we administered i.v. N-7DHC-lipos (10 mg/kg) or PBS to healthy C57BL/6 mice and collected blood for testing after 14 days (Fig. 5f). Biochemical markers including blood urea nitrogen (BUN) and alanine transaminase (ALT), which are measures of liver and kidney function, remain in the normal range (Fig. 5g). The complete blood count (CBC) also showed no difference between samples from the N-7DHC-lipos and phosphate-buffered saline (PBS) groups for all indices (Fig. 5h, Table S1). Histopathology on major organ tissues including lung, liver, kidney and spleen, and performed histopathology also revealed no evidence of toxicity (Fig. 5i). Taken together, the data suggest that N-7DHC-lipos are well tolerated by the animals.

### Radiosensitizing effects of N-7DHC-lipos in vivo

**Fig. 6:**
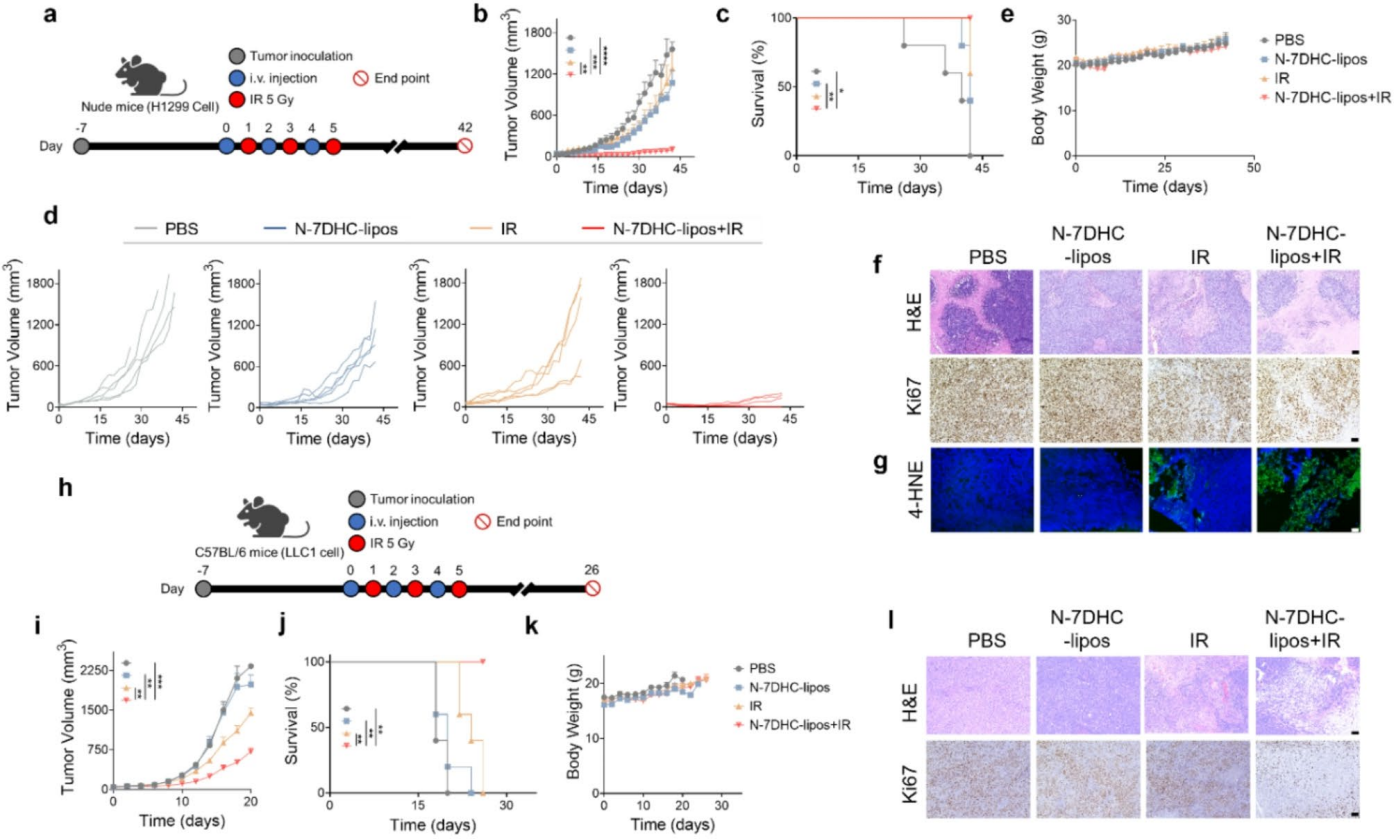
Therapeutic efficacy of N-7DHC-lipos, which was evaluated in both H1299 tumor-bearing nude mice and LLC1 tumor-bearing C57BL/6 mice. (**a**) Scheme of efficacy studies conducted in H1299 tumor-bearing nude mice. 7-DHC-lipos (10 mg/kg) were injected i.v., followed by 5 Gy tumor irradiation at 24 h. A total of three treatments were administered every two days (*n* = 5 mice). For comparison, PBS alone, 7-DHC-lipos alone, and IR alone were tested. (**b**) Average tumor growth curves. (**c**) Animal survival curves. (**d**) Individual tumor growth curves. (**e**) Average animal body weight curves. (**f**) H&E and Ki67 staining of tumor tissue samples. Scale bars, 200 μm. (**g**) Immunofluorescence microscopy of tumor tissue stained for 4-HNE (*n* = 3 mice). H1299 tumor-bearing mice were treated with a dose of 7-DHC-lipos plus IR, and tumor samples were collected after euthanasia 24 h after IR. Scale bar, 50 μm. (**h**) Scheme of efficacy studies performed in LLC1 tumor-bearing C57BL/6 mice. The same nanoparticle and irradiation dose were used as in the H1299 study (*n* = 5 mice). (**i**) Average tumor growth curves. (**j**) Animal survival curves. (**k**) Average animal body weight curves. (**l**) H&E and Ki67 staining of tumor tissue samples from different treatment groups. Scale bars, 200 μm. Data are presented as mean ± SD. Statistical difference was evaluated using one-way ANOVA for tumor volume in (b, i), and Mantel-Cox test for survival in (c, j). *p < 0.05, **p < 0.01, ***p < 0.001, ****p < 0.0001

We subsequently evaluated the efficacy of the NTS-7DHC-lipos to enhance radiotherapy in vivo in the same H1299 model. We administered i.v. N-7DHC-lipos (10 mg/kg) to the animals and irradiated the tumors (5 Gy) after 24 h (*n* = 5 mice). A total of three treatments were administered every two days (Fig. 6a). H1299 cancer cells are known to be relatively resistant to radiotherapy [47–49], and irradiation alone only moderately suppressed tumor growth (Fig. 6b). N-7DHC-lipos alone slightly slowed down tumor growth but the difference was not significant (*p* = 0.074 on Day 26). In comparison, the combination of N-7DHC-lipos and irradiation significantly improved the treatment outcome. The tumor inhibition rates for the combination were 94.4% on Day 26 an increase of more than 55% compared to irradiation alone. At the end of the experiment on Day 42, all mice in the combination treatment group were alive and 40% of them remained tumor free (Fig. 6b-d). In comparison, 40% of the animals in the irradiation-only group had either died or reached a humane endpoint.

Post-mortem H&E and Ki67 staining of tumor sample sections confirmed a reduced mass density and decreased proliferation in tumors treated with the combination (Fig. 6f). To examine early tumor response, in a separate study, we treated H1299 mice with N-7DHC-lipos and irradiation and euthanized the animals after 24 h for 4-HNE staining. We found a significantly increased level of positive 4-HNE staining in tumors from the 7DHC-lipos plus irradiation group, validating that the combination effectively induced ferroptosis (Fig. 6g).

In addition to the H1299 model, we also evaluated the radiosensitizing effects of N-7DHC-lipos in C57BL/6 mice bearing LLC1 tumors (Fig. 6h). LLC1 is a highly aggressive tumor and irradiation alone showed limited therapeutic benefit (Fig. 6i). In contrast, the combination of N-7DHC-lipos and irradiation significantly reduced tumor growth (Fig. 6i, S7). Animal survival was also significantly improved (Fig. 6j). Post-mortem H&E and Ki67 staining supported the enhanced cancer cell killing with the combination (Fig. 6l). Importantly, no acute toxicity or significant weight loss was observed throughout the therapeutic studies in either animal model (Fig. 6e&k, Fig. S8&9).

## Conclusion

In this study, we have demonstrated that 7DHC-encapsulated liposomes can act as an inducer of ferroptosis. The ability of 7DHC to elicit ferroptosis is fully realized only when used in combination with irradiation, which set it apart from conventional ferroptosis inducers. In addition, unlike current inducers that mainly target the xCT/GSH/GPX4 axis, 7DHC directly targets unsaturated lipids, causing damage to cell membranes. In addition, 7DHC produces a variety of oxysterol and aldehyde by-products upon irradiation, which may contribute to or enhance the radiation-induced bystander effect [50, 51], thereby augmenting the efficacy of radiotherapy.

The ability of liposomes to incorporate 7DHC in a manner similar to cholesterol and partially fuse with cell membranes is conducive to radiation-induced ferroptosis. The use of external stimuli to induce ferroptosis results in improved tumor selectivity and reduced systemic toxicity. Indeed, our results show that the therapeutic benefit of N-7DHC-lipos is only manifested within the tumor upon irradiation. CBC and histopathology data found no detectable side effects with the nanoparticles, which is likely because 7DHC is safely metabolized to cholesterol in normal tissues outside the radiation field.

The technology is highly clinically relevant, given the wide use of radiotherapy in cancer treatment. For example, in non-small cell lung cancer, approximately one-third of patients receive radiotherapy at least once during their treatment regimen [52]. To enhance efficacy within the limits of safe radiation doses, radiosensitizers are commonly employed in conjunction with radiotherapy, i.e. chemoradiotherapy. Conventional radiosensitizers, such as cisplatin, carboplatin, and paclitaxel, have demonstrated their ability to improve the effectiveness of radiotherapy. However, these agents are frequently associated with significant systemic toxicity [53, 54], rendering the combination therapy intolerable for many patients. Furthermore, acquired resistance to chemotherapy, which primarily targets DNA or DNA repair mechanisms, remains a major challenge. These limitations highlight the urgent need for the development of radiosensitizers with alternative mechanisms of action. However, while ferroptosis inducers have shown promise in enhancing radiosensitization, their in vivo application may be limited due to challenges in selectively delivering sufficient quantities of these agents to tumors. Our approach, which allows for targeted induction of ferroptosis within tumors under radiation, addresses this challenge.

In this study, we primarily used a fraction dose of 5 Gy, a standard approach in preclinical experiments. Future studies will explore alternative fractionation and dosing strategies to optimize therapeutic outcomes. While our histological and biochemical analyses demonstrated minimal toxicity with a single injection of N-7DHC-liposomes, further research is required to assess the effects of repeated dosing and evaluate potential long-term toxicity. For successful clinical translation, several challenges must be addressed, including large-scale synthesis, stability under physiological conditions, and regulatory compliance. Additionally, while oxysterols are known to be metabolized by P450 enzymes, the specific metabolic pathways and enzymes involved vary by oxysterol type and tissue context. Detailed mechanistic studies to elucidate the metabolism of oxysterols generated in this system would be valuable. We aim to address these critical issues in future investigations.

## Methods

### Materials

Phosphatidyl choline (L-α-phosphatidylcholine, 95%, Avanti Polar Lipids, Cat# 131601), triolein (glyceryl trioleate, Sigma, Cat# T7140), 7-dehdrocholesterol (Sigma, Cat# 30800), cholesterol (Sigma, Cat# C3045), chloroform (Fisher Scientific, C298-4), methanol (Fisher Scientific, A452-4), 100-nm filter (Whatman, 800309), trypsin (0.25% with EDTA, Corning, Cat# 25-053-CI), DMSO (dimethyl sulfoxide, Fisher Scientific, Cat# BP231-1), trypan blue (0.4% in PBS, Corning, Cat# 25-900-CI), PBS (phosphate buffer saline, pH 7.4, Gibco, Cat# 10010023), Milli-Q Water (H_2_O, 18.2 MΩ.cm@25°C), crystal violet (Sigma, Cat# C0775), paraformaldehyde (4% in PBS, Chem Cruz, Cat# sc-281692), acetone (Fisher Scientific, A18-4), formalin (10% neutral buffered, Cancer Diagnostics, Cat# FX1000), sodium azide (Sigma, Cat# S8032), dynasore (Sigma, Cat# D7693), nystatin (Sigma, Cat# N6261), chlorpromazine (chlorpromazine hydrochioride, Sigma, Cat# C0982), tocopherol (α-tocopherol polyethylene glycol succinate, TCI, Cat# T3118), ascorbic acid (L-ascorbic acid, Sigma, Cat# A92902), ferrostatin-1 (Cayman, Cat# C816Z13), deferoxamine (deferoxamine mesylate, Sigma, Cat# PHR3411), glycine (Sigma, Cat# G7126), Z-VAD-FMK (Sigma, Cat# V116), 3-methyladenine (Sigma, Cat# M9281). Myristoylated NTS_mut_ was custom-ordered from CSBio.

### Nanoparticle Preparation

Phosphatidyl choline, triolein and 7-dehydrocholesterol (7DHC) were dissolved in a CHCl_3_/MeOH (v: v 2:1) solvent at a 3:2:1 molar ratio. For preparation of N-7DHC-lipos, myristoylated NTS_mut_ (Lys-Pro-(NMe-Arg)-Arg-Pro-Tyr-Tle-Leu) at a 1:20 molar ratio to phosphatidylcholine was also added to the mixture. After removing the solvent via rotary evaporation, Tris buffer (pH = 8.0) was added to the flask and stirred for an hour at 55–65 °C to reconstitute the nanoparticles. The nanoparticles were then extruded at 55–65 °C through a 100-nm filter using a mini-extruder (Avanti Polar Lipids, Cat# 610020) for size uniformity. The resulting nanoparticles were stored at 4 °C. Synthesis of cholesterol encapsulated liposome counterpart followed the same procedure except replacing 7DHC with cholesterol.

To analyze targeting ligand and 7DHC contents, liposomes were lyophilized, weighted, and reconstituted in MeOH. The concentrations of myristoylated NTS_mut_ and 7DHC was determined using LC-MS (Bruker Elute UHPLC and Bruker Impact II) with the following settings: Chromatography (Mobile Phase: 90% methanol, 10% water, 0.1% formic acid; Flow rate: 0.4 mL/min; 15 min isocratic gradient; Column Temperature: 40 °C); Column (Kinetex, Evo C18, 1 × 100 mm, 1.7 μm, 100 Å); Mass Spectrometry (Positive ionization mode (ESI); Voltage: 2.5 kV; Desolvation Temperature: 450 °C; Desolvation Gas Flow: 700 L/hr). Standards for 7DHC and myristoylated-NTS_mut_ were analyzed to establish standard curves, which were used to quantify the concentrations of 7DHC and myristoylated-NTS_mut_ in the samples.

### Cryogenic TEM

Cryogenic TEM (cryo-TEM) grids were prepared using Vitrobot Mark IV (Field Electron and Ion Company, Hillsboro, OR) with the following settings: blot force of -10, wait time of 10 s, and blot times of 3,4 and 6 s. Quantifoil R1.2/1.3 400 Cu mesh grids were rendered hydrophilic with a TergeoEM (PIE Scientific LLC, Union City, CA) using indirect oxygen-argon plasm (25:75 ratio). 7DHC-Lipos or N-7DHC-Lipos solutions (~ 10 mg/mL) were applied to the carbon side of a TEM grid prior to vitrification by immersion in liquid ethane-propane (40:60 mixture). All images were analyzed using Image J software with at least 50 nanoparticle measurements to ensure a global representation of the assembled structure.

### Dynamic light scattering and zeta potential

Zeta potential and size distribution measurements were carried out using a Malvern Zetasizer Nano ZS system. Prior to analysis, the solvent was exchanged for 1× PBS (pH 7.4) using a desalting column. To evaluate the stability of the liposomes, samples were stored at 4 °C for one week and the dynamic light scattering (DLS) was performed on days 1, 2, 3, and 7.

### 7DHC release

To measure 7DHC release, 7DHC-Lipos were loaded into a 10k MWCO dialysis tube and the tube was immersed in PBS solutions with pH values of 5.5, 6.5, and 7.4, respectively. The solutions were mounted on a shaker and the incubation temperature was maintained at 37 °C. Aliquots of the samples were taken at different time points (0.25, 0.5, 1, 2, 4, 8, 12, 24, 48 and 72 h), and the 7DHC content was quantified by LC-MS, as described above. For CHOL-Lipos, released cholesterol was quantified using a Cholesterol Quantification Assay kit (Sigma, Cat# CS0005) following the manufacturer’s instructions.

### Cell culture

NCI-H1299, Hcc827, H460, and LLC1 cells were purchased from ATCC and cultured according to ATCC protocols. Typically, a complete growth medium was prepared by adding 50 mL fetal bovine serum (FBS, Atlanta Biologicals, Cat# S11150) and 5 mL penicillin-streptomycin (Corning Cat# 30-002-CI) to 445 mL of RPMI 1640 medium (Corning, Cat# 10-104-CV). Cells were subcultured every three days and maintained at 37 °C in a Thermo Scientific Heracell 150i incubator. One day before the experiment, the cells were washed with PBS, trypsinized (37 °C, 2 min), neutralized with cell culture medium, and centrifuged (1200 rpm, 5 min). The supernatant was removed, and the cells were resuspended in a fresh cell culture medium. For viability and other assays, cells were quantified using a hemocytometer (Hausser Scientific, Cat# 3200) before seeding the desired number of cells onto plates.

### Cellular uptake (Florescence Microscopy)

DiR dye (Biotium, Cat# 60017) was added during the synthesis of 7DHC-Lipos and N-7DHC-Lipos, and was incorporated into the lipid layer of the nanoparticles. One day before the incubation, 1 × 10^5^ H1299 cells were seeded on a 2-chamber glass slide (Nunc™ Lab-Tek™ II Chamber Slide™ System, ThermoFisher, Cat# 154534) and incubated at 37 °C overnight. The cell culture medium was removed, and the cells were incubated with 1 mL of serum-free cell culture medium containing DiR-labeled 7DHC-Lipos/N-7DHC-Lipos (50 µg/mL) for 4 h at 37 °C. After incubation, the cells were washed three times with PBS, fixed with 4% paraformaldehyde, and stained with DAPI. Fluorescence images were captured with a fluorescence microscope (Keyence, BZ-X800). To examine the ability of N-7DHC-Lipos to fuse with cell membranes, the nanoparticles were labeled with both calcein (Cayman, Cat# 16221) and DiL (ThermoFisher, Cat# D3911), which were loaded into the interior and the lipid layer of the nanoparticles, respectively. The nanoparticles were incubated with H1299 cells and the live cells were imaged by fluorescence microscopy. The images were analyzed using Image J.

### Cellular uptake (flow cytometry)

16:0 Liss Rhod PE (Avanti, Cat# 810158 C) was added during the synthesis of 7DHC-Lipos and N-7DHC-Lipos to dye-label the nanoparticles. One day before incubation, 5 × 10^5^ H1299 cells were seeded on a 6-well plate (Corning, Cat# 3516) and incubated at 37 °C overnight. The cell culture medium was removed, and the cells were incubated for with 2 mL of Rhod-labeled 7DHC-Lipos or N-7DHC-Lipos suspended in serum-free cell culture medium (50 µg/mL). For comparison, endocytosis inhibitors, including sodium azide (50 mM), dynasore (80 µM), nystatin (25 µM), and chlorpromazine (100 µM), were co-incubated with the nanoparticles. After 4 h, the cells were washed once with PBS, then incubated with DAPI-containing staining buffer (ThermoFisher, Cat# D1306) for 5 minutes. Next, the cells were collected by a cell lifter and washed once with PBS. Cells were then fixed with a 1:1 mixture of IC fixation buffer (Invitrogen, Cat# 00-8222-49) for 15 min, and resuspended in staining buffer. Trypan blue solution (20 µg/mL) was added to quench fluorescence from nanoparticles non-specifically bound to cell membranes. Flow cytometry was performed on NovoCyte Quanteon Flow Cytometer Systems (Agilent) and the mean fluorescence intensity (MFI) of Rhod was recorded. The uptake study with NTSR1 negative Hcc827 cells followed the same protocol.

### Cell binding assay

The affinity of N-7DHC-Lipos for NTSR1 was assessed using neurotensin (NTS)-NOTA-Cu^64^ as a competitive ligand. Briefly, H1299 cells were seeded in a 24-well plate (1.5 × 10^5^ cells per well) and incubated overnight at 37 °C in a 5% CO_2_ atmosphere. The medium was replaced with serum-free medium containing different concentrations of N-7DHC-Lipos (with NTS_mut_ concentration of 400, 200, 50, 20, 2, and 0.2 nM, respectively) and 5 µCi of NT-NOTA-Cu^64^. For comparison, solutions containing free NTS (10000, 1000, 100, 10, and 1 nM, CSBio, CA) and 5 µCi of NT-NOTA-Cu^64^ were tested. All concentrations were tested in triplicate. After incubation for 1 h at 37 °C in a 5% CO_2_ atmosphere, the cells were rinsed three times with ice-cold PBS, and incubated with 1 N NaOH. Cells were collected and their radioactivity was measured using a γ-counter (PerkinElmer). Data were analyzed using Prism (GraphPad). Figures were plotted as counts per minute of radioactivity versus the concentration of NTS in nM on a log scale.

### Cellular superoxide and hydroxyl radical levels

Superoxide levels were assessed using the Dihydroethidium Assay Kit (DHE, ThermoFisher, Cat# D11347). Briefly, H1299 cells (8,000 cells per well) were seeded on a black 96-well plate (Corning Costar, Cat# 3603) overnight. The next day, the cell culture medium was replaced with fresh serum-free medium containing either PBS or 50 µg/mL of N-7DHC-Lipos. After incubation for 4 h at 37 °C, the medium was removed and replaced with 5 µM DHE in FBS-free RPMI medium. After incubation in the dark for 30 min at room temperature, the cells were irradiated (5 Gy, X-Rad 320 Irradiator). DHE fluorescence (ex/em: 518/605 nm) was measured using a microplate reader (Synergy Mx, BioTeK). In control groups, nystatin (25 µM), tocopherol (100 µM), or ascorbic acid (100 µM) were added together with N-7DHC-Lipos to incubate with the cells.

Hydroxyl radical levels were assessed using the Aminophenyl Fluorescein Assay Kit (APF, Invitrogen™ Cat# A36003) by reading APF fluorescence (ex/em: 490/515 nm). Otherwise, the protocol was the same as for the DHE study.

### Superoxide dismutase (SOD) activity

H1299 cells were seeded in a 6-well plate at a density of 1 × 10^6^ cells per well (Corning, Cat# 3516) and incubated overnight at 37 °C. The next day, the cell culture medium was replaced with fresh serum-free medium containing either PBS or 50 µg/mL of 7DHC-Lipos. After incubation for 4 h at 37 °C, the cells were irradiated (5 Gy) using an X-RAD 320 irradiator. After incubation for an additional 1 h, the cells were washed 3 times with PBS, and collected with a cell scraper followed by centrifugation (1200 x g, 5 min). The cells were re-suspended in 1 mL of PBS and lysed by sonication with a probe sonicator (Fisherbrand™ Model 120 Sonic Dismembrator) in an ice bath (30% amplitude, 30 s, 5 s pause every 10 s). The supernatant was collected by centrifugation (1500 x g, 5 min) and analyzed with the Superoxide Dismutase Assay Kit (Cayman Chemical, Cat# 706002) according to the manufacturer’s protocol. Absorbance at 440 nm was measured using a microplate reader (Synergy Mx, BioTeK).

### DNA damage

H1299 Cells (1 × 10^6^) were seeded in a 6-well plate and incubated overnight. The next day, the cell culture medium was replaced with fresh serum-free medium containing PBS or 50 µg/mL of N-7DHC-Lipos. For the IR and 7DHC-Lipos plus IR groups, the cells received 5 Gy irradiation after 4 h. After further incubation for 1 h in full growth medium, cells were collected, fixed, permeabilized, and stained anti-γH2AX (Alexa 488) antibody (Biolegend, Cat# 613405) and DAPI according to the manufacturer protocol. The stained cells were analyzed using flow cytometry. In control groups, cells were co-incubated with nystatin (25 µM), tocopherol (100 µM), or ascorbic acid (100 µM) for comparison.

### Lipid peroxidation

Lipid peroxidation was assessed using BODIPY™ 581/591 C11 (Lipid Peroxidation Sensor, ThermoFisher, Cat# D3861). Briefly, H1299 cells (8,000 cells per well) were seeded onto a black 96-well plate (Corning Costar, Cat# 3603). The next day, the cell culture medium was replaced with serum-free medium containing PBS or 50 µg/mL of N-7DHC-Lipos. After incubation at 37 °C for 4 h, the medium was replaced with 5 mM BIDOPY in serum-free RPMI medium, and the incubation continued in the dark for 30 min. Then, for the IR and 7DHC-Lipos + IR groups, cells were irradiated with 5 Gy. Red (ex/em: 581/590 nm) and green (ex/em: 488/510 nm) fluorescence was immediately measured using a microplate reader (Synergy Mx, BioTeK), and the green/red fluorescence ratio was calculated.

### Cellular MDA and 4-HNE levels

Briefly, H1299 cells (10^6^ cells per dish) were seeded onto 100 mm cell culture dishes (Corning, Cat# 353003). The next day, the cell culture medium was replaced with fresh serum-free medium containing either PBS or 50 µg/mL of 7DHC-Lipos and incubated at 37 °C for 4 h. For the IR and 7DHC-Lipos + IR groups, the wells were irradiated (5 Gy), then all cells were further incubated in full growth medium for 24 h. Cells were collected with a cell scraper, resuspended in 1 mL PBS, and lysed with a probe sonicator in an ice bath. The supernatant was collected by centrifugation (1500 x g, 5 min). The contents of 4,4’-methylenebisbenzenamine (MDA) and 4-Hydroxynonenal (4-HNE) were quantified using a TBARS Assay Kit (Cayman Chemical, Cat# 100009055) and a 4-HNE Assay Kit (abcam, Cat# ab238538), respectively.

### Cellular sterol and oxysterol levels

Cell samples were prepared using the same protocol as for the MDA and 4-HNE studies. Lipid extraction and UHPLC-MS/MS were performed according to published protocols [55, 56]. Briefly, cell samples were lysed in an ice-cold ultrasonic bath for 30 min and vortexed. The protein content of each sample was quantified using the BioRad-DC Protein Assay Kit. Isotope-labeled internal standards (d_7_-cholesterol, d_7_-7-dehydrocholesterol, ^13^C_3_-desmosterol, and ^13^C_3_-lanosterol for sterols, and d_7DHC_EO, d_7_-7-keto-cholesterol, d_6_-24,25-epoxycholesterol, d_7_-24-hydroxycholesterol, and d_7_-4β-hydroxycholesterol for oxysterols) were added to each sample. 1 mL of 0.9% NaCl aqueous solution and 4 mL of Folch solution (2:1 *v/v* CHCl_3_: MeOH, with 1 mM BHT and 1 mM PPh_3_) were also added. The samples were vortexed for 30 s. After centrifugation, the lower organic layer was extracted and dried down in a speed vacuum concentrator. Samples were reconstituted in methylene chloride and stored at -80 °C until analysis. Sterol and oxysterol analysis was performed by UHPLC-MS/MS on an AB Sciex Triple Quad 6500 instrument. Samples were prepared at 2:1 concentration before injection. Data were analyzed using Analyst software. Protein content was used for data normalization. The mass spectroscopy studies were performed at the Mass Spectrometry Center, School of Pharmacy, University of Washington.

### Cytotoxicity and clonogenicity

Cell viability was assessed with H1299 cells using the standard MTT assay. Briefly, H1299 cells (4,000 cells per well) were seeded on a 96-well plate (Corning Costar, Cat#3599). The next day, the cell culture medium was replaced with fresh serum-free medium containing either PBS or 50 µg/mL of 7DHC-Lipos and incubated at 37 °C for 4 h. For the IR and 7DHC-Lipos + IR groups, the wells were irradiated (5 Gy), then all cells were further incubated in full growth medium for 48 h. Twenty µL of 10 mg/mL 3-(4,5-Dimethylthiazolyl-2)-2,5-diphenyltetrazolium bromide (MTT) solution was added to each well, followed by incubation at 37^o^C for 4 h. After incubation, the MTT solution was replaced with 100 µL of DMSO to solubilize the purple formazan crystals. Absorbance at 570 nm was measured using a microplate reader (Synergy Mx, BioTeK). For comparison, Ferr-1 (10 µM), DFO (5 µM), Z-VAD (0.1 mM), Gly (5 µM), or 3-MA (5 mM) were added together with the nanoparticles to incubate with cells.

For the LDH release assay, the cells were treated according to the same protocol as above, except that the incubation was stopped 24 h after the end of irradiation. For the total LDH group, 10 µL lysis buffer was first added to the incubation medium and incubated with cells at 37 °C for 30 min to release all the LDH. Then, for all treatment groups, 100 µL of supernatant was transferred to a new transparent 96-well plate and mixed with 100 µL of LDH kit working solution. The plate was incubated at room temperature for 30 min, followed by the addition of 50 µL of LDH kit working solution to stop the reaction. The absorbance at 570 nm was measured using a microplate reader (Synergy Mx, BioTek), and the percentage of LDH release was calculated by comparing the absorbance of each group to the total LDH group.

The ATP release was measured using the ATPlite 1step Luminescence Assay Kit (PerkinElmer, Cat# 6016731) according to the manufacturer’s protocol. Cells were treated according to the same protocol as for the LDH assay. After 24 h, 100 µL of supernatant from each well was transferred to a white 96-well plate (Corning Costar, Cat# 3610), followed by the addition of 70 µL of the ATP kit solution. Luminescence signals were measured immediately using a microplate reader (Synergy Mx, BioTek) and compared to a pre-established calibration curve to derive ATP concentrations.

For clonogenicity assay, H1299 cells were incubated with either PBS or 7DHC-Lipos for 4 h at 37 °C, and the dissociated cells were seeded onto a 100 mm cell culture plate (Corning, Cat# 353003) at a density ranging of 100 to 10,000 cells. The cells were then irradiated with a dose range of 0–10 Gy. After 14 days, cell colonies were stained with crystal violet and counted, and a survival fraction (*S*) relative to the untreated control was calculated. The data were fitted into the LQ model: $\:S={e}^{-(\alpha\:D+\beta\:{D}^{2})}$. A clonogenic assay was also performed to evaluate ferroptosis. In that case, H1299, H460, and H226 cells were treated with 7DHC-Lipos plus 5 Gy irradiation, with or without Ferr-1 (10 µM).

### Western blot

H1299 cells (0.5 M cells per well) were seeded in 6-wells plate overnight, then cell culture medium was replaced with fresh serum-free medium containing either PBS or 50 µg/mL of 7DHC-Lipos and incubated at 37 °C for 4 h. For the IR and 7DHC-Lipos + IR groups, the wells were irradiated (5 Gy), then all cells were further incubated in full growth medium for 24 h. The total cell proteins were extracted in RIPA lysis buffer (Thermo Scientific, Cat# 89901) supplemented with 1× proteinase inhibitor cocktail (Thermo Scientific, Cat# 78445), then quantified with BCA protein assay kit (Thermo Scientific, Cat# PI23225), resolved in a 10–12% SDS-PAGE gel, and then were incubated with appropriate primary antibodies. This is followed by incubation with secondary antibodies and exposed to X-ray films (Santa Cruz, Cat# 201696). The antibodies used are: KEAP1 (CST, Cat# 8047); GPX4 (CST, Cat# 52455); SLC7A11 (CST, Cat# 12691); SLC11A2 (CST, Cat# 15083); ASCL4 (Abcam, Cat# ab205197); GAPDH (CST, Cat# 2118); Apoptosis Western Blot Cocktail (Abcam, Cat# ab136812); Anti-rabbit IgG, HRP-linked Antibody (CST, Cat# 7074); Anti-mouse IgG, HRP-linked Antibody (CST, Cat# 7076).

### TEM of cell samples

H1299 cells were treated with N-7DHC-Lipos (50 µg/mL) plus irradiation (5 Gy) as described above. Cells were then harvested and processed as previously described with some modifications [57]. Briefly, cells were fixed overnight with a solution containing 3% glutaraldehyde and 2% paraformaldehyde in 0.1 M cacodylate-HCl buffer, pH 7.25 at 4 °C. After being washed several times in 0.1 M cacodylate-HCl buffer solutions, the cells were agar-enrobed with 3% Noble Agar at 60 °C. After cooling, agar-cell pellets were extracted from Eppendorf tubes and placed in 0.1 M cacodylate-HCl buffer for further processing. Cells were then treated with 0.1% Millipore-filtered cacodylate-buffered tannic acid (30 min) and rinsed well in 0.1 M cacodylate-HCl buffer, pH 7.25. Cells were postfixed with 1% buffered osmium (1 h), rinsed well, and stained en bloc with 1% Millipore-filtered uranyl acetate (1 h in the dark). After rinsing well in deionized water, the cells were dehydrated in increasing concentrations of ethanol, infiltrated with propylene oxide, and embedded in an Epon-Araldite plastic [58]. Embedded cell pellets were polymerized in a 60 °C oven for 3 days. Ultrathin sections were cut on a Reichert Ultracut S ultramicrotome, placed on clean 200-mesh Cu Hex grids, and stained with uranyl acetate and lead citrate. Sections were examined on a JEOL JEM-1011 transmission electron microscope (JEOL USA, Inc.) at an accelerating voltage of 100 kV. Digital images were acquired using an AMT Imaging System (Advanced Microscopy Techniques). All processing, sectioning, and imaging was performed at the Georgia Electron Microscopy Core Facility on the campus of the University of Georgia.

### In vivo whole-body fluorescence imaging

All animal experiments were conducted in accordance with an Animal Use Protocol (AUP) approved by the University of Georgia Institutional Animal Care and Use Committee (IACUC, PHS Assurance No. D16-00276). The in vivo imaging study was performed in nude mice bearing flank H1299 tumors. Briefly, 5 × 10^6^ H1299 cells were injected subcutaneously into the right flank of a female 4–6-week-old female mouse (Charles River). When the tumor size reached 300 mm^3^, 10 mg/ml of DiR-labeled N-7DHC-Lipos or 7DHC-Lipos were injected intravenously into each mouse (*n* = 3 mice). Whole-body fluorescence images were acquired on a Vivo & In Vitro Imaging scanner (NEWTON 7.0) at 0.5, 4, and 24 h after injection. After 24 h, the tumors and major organs, including the liver, lung, brain, muscle, and kidney were harvested and scanned ex vivo. ROI analysis was performed to assess the distribution of nanoparticle in the tissues. Tumor samples were embedded in O.C.T. compound and then frozen at -80 °C. Tumor slices of 8 μm thickness were sectioned on a cryostat, which were then fixed with acetone, and stained with DAPI. Microscopic images were taken on a fluorescence microscope (Keyence, BZ-X 810).

### Hematology and blood biochemistry

Healthy BALB/C mice (4–6 weeks old, Envigo) were injected intravenously with PBS or N-7DHC-Lipos (10 mg/kg, 50 µL) (*n* = 3 mice) and were euthanized after 14 days. Blood samples were collected by cardiac puncture. Major organs including liver, heart, lung, kidney, spleen, and intestine were harvested. Complete blood count (CBC) and histopathology were performed at Clinical Pathology Lab, College of Veterinary Medicine, University of Georgia. Liver and kidney functions were assessed using the Alanine Aminotransferase (ALT) Kit (Abcam, Cat# ab105134) and the Urea Nitrogen (BUN) Colorimetric Detection Kit (ThermoFisher, Cat# EIABUN) according to the manufactures’ protocols.

### In vivo radiation therapy study

The efficacy study was evaluated in both H1299 and LLC-1 flank tumor models. The H1299 model was established by subcutaneous injection of 5 × 10^6^ H1299 cells into the right flank of 4-week-old female nude mice. LLC-1 model was established by subcutaneous injection of 1 × 10^6^ into the right flank of 4-week-old C57BL/6 mice. All animals were obtained from Envigo. When the tumor size reached 50 mm^3^, the mice were randomly divided into four groups (*n* = 5 mice). Animals in the 7DHC-Lipos + IR group were injected i.v. with N-7DHC-Lipos (10 mg/kg in 50 µL PBS). After 24 h, the animals received tumor irradiation (5 Gy), while the rest of the animal body was lead-shielded. In control groups, animals were treated with PBS alone, N-7DHC-Lipos alone, or IR alone. Two additional treatments were administered two days apart. Tumor size was measured every two days using a caliper, and tumor volume was calculated using the equation: $\:Tumor\:volume=\frac{tumor\:length\:x\:{tumor\:width}^{2}}{2}$), where tumor length ≥ tumor width. Mice were euthanized when they reached a humane endpoint such as length greater than 1.7 cm, weight loss more than 20%, or the presence of any tumor discharge. Tumors and major organs such as liver, heart, lung, kidney, spleen, and intestine were collected. Hematoxylin and eosin (H&E) and Ki67 staining were performed at the Histology Laboratory, College of Veterinary Medicine, University of Georgia. The microscopic images were captured with a digital microscope (Keyence, BZ-X 810).

In separate animals (H1299 bearing nude mice, *n* = 3 mice), animals from the four treatment groups were euthanized 24 h after single dose of treatment. Tumor tissue sections were stained with anti-4-hydroxynonenal antibody (Sigma, AB5605) according to a published protocol [59]. Microscopic images were taken under a fluorescence microscope (Keyence, BZ-X 810).

### Statistical analysis

The means and standard deviations were calculated from at least three replicate groups in all the experiments. Statistical significance was calculated by one-way ANOVA with post-hoc Tukey-Kramer comparisons (for more than two groups) or two-tailed Student’s t test (for two groups). P values less than 0.05 were considered statistically significant. *, *p* < 0.05; **, *p* < 0.01; ***, *p* < 0.001; ****, *p* < 0.0001; ns, *p* > 0.05.

## Electronic supplementary material

Below is the link to the electronic supplementary material.

Supplementary Material 1: **Supporting Information**. The online version contains Supplementary Information, which include Supplementary Figures. Supplementary **Figure S1**– **S9** show physicochemical characterization of N-CHOL-lipos, influence of a micropinocytosis inhibitor on cellular uptake of N-CHOL-lipos, membrane fusion of N-CHOL-lipos, comparison between N-CHOL-lipos and N-7DHC-lipos in terms of inducing oxidative stress increase and DNA damage, proposed mechanism for 7DHC-induced autooxidation under radiation, cell toxicity of N-7DHC-lipos, individual tumor growth curves, and H&E staining results. Supplementary **Table S1** shows CBC results

## Data Availability

No datasets were generated or analysed during the current study.
