# Supplementary material for: Radiation-induced ferroptosis via liposomal delivery of 7-Dehydrocholesterol
Source: J Nanobiotechnology. 2025 Mar 26;23:249. doi: 10.1186/s12951-025-03303-3 (PMC11938788; doi:10.1186/s12951-025-03303-3)
Supplement: Supplementary file 1 — Supplementary Material 1: Supporting Information. The online version contains Supplementary Information, which include Supplementary Figures. Supplementary Figure S1– S9 show physicochemical characterization of N-CHOL-lipos, influence of a micropinocytosis inhibitor on cellular uptake of N-CHOL-lipos, membrane fusion of N-CHOL-lipos, comparison between N-CHOL-lipos and N-7DHC-lipos in terms of inducing oxidative stress increase and DNA damage, proposed mechanism for 7DHC-induced autooxidation under radiation, cell toxicity of N-7DHC-lipos, individual tumor growth curves, and H&E staining results. Supplementary Table S1 shows CBC results [file 12951_2025_3303_MOESM1_ESM.pdf]

Supporting Information for

Radiation-Induced Ferroptosis via Liposomal Delivery of 7-Dehydrocholesterol

Jianwen Li[1], Shuyue Zhan[1], Wei Yang[1], He Zhang[2], Xinrui Ma[2], Fanghui Chen[3], Amy Li[4], Pakteema Tong[4], Fangchao Jiang[1], Zhengwei Cao[1], Ian Delahunty[1], Jiayi Wang[1], Yufei Wu[1], Zhi Liu[1], Zibo Li[2], Yong Teng[3], Libin Xu[4], and Jin Xie[1]*

[1]Department of Chemistry, University of Georgia, Athens, GA 30602, USA.

[2]Department of Radiology, University of North Carolina at Chapel Hill, Chapel Hill, NC 27599, USA.

[3]Department of Hematology and Medical Oncology & Winship Cancer Institute, Emory University School of Medicine, Atlanta, GA 30322, USA.

[4]Department of Medicinal Chemistry, University of Washington, Seattle, WA 98195, USA.

Corresponding Author

*Jin Xie - Department of Chemistry, University of Georgia, Athens, Georgia 30602, United States; Email: jinxie@uga.edu

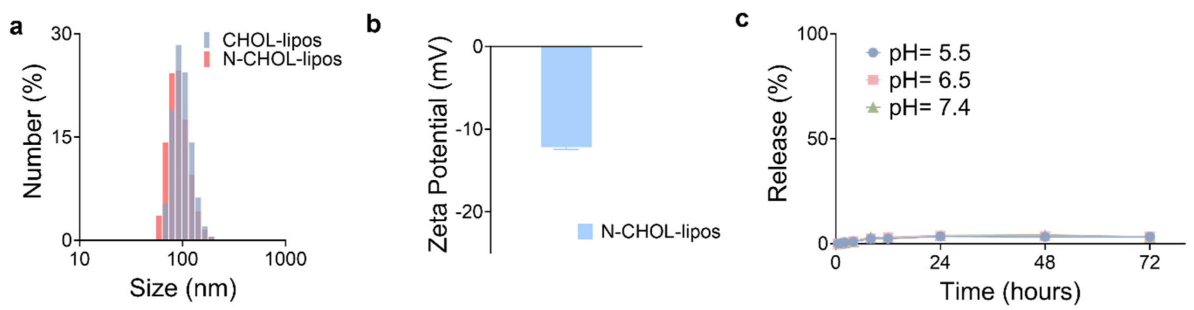

Fig. S1 Physicochemical characterization of N-CHOL-lipos. a) Hydrodynamic sizes of cholesterol-encapsulated liposome nanoparticles (CHOL-lipos) and NTS_{mut}-conjugated and cholesterol-encapsulated liposome nanoparticles (N-CHOL-lipos). b) Zeta potentials of N-CHOL-lipos in PBS. c) Cholesterol release from CHOL-lipos, tested in PBS solutions with pH at 7.4, 6.5, and 5.5.

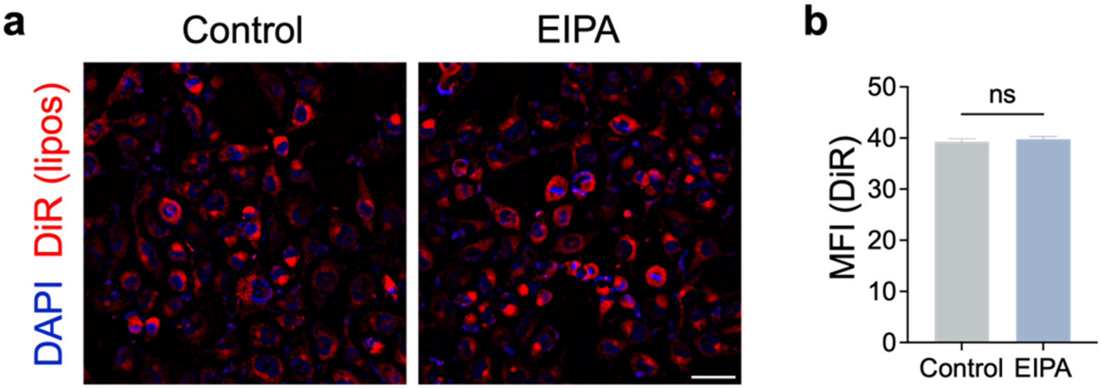

Fig. S2 (**a**) Fluorescence microscopy evaluating DiR-labeled N-7DHC-lipos uptake by H1299 cells using fluorescence microscopy, in the presence of absence of macropinocytosis inhibitor EIPA (5-(N-Ethyl-N-isopropyl)amiloride). Scale bar, 50 μm. (**b**) Mean fluorescence intensity (MFI) of DiR in cells, analyzed by Image J based on microscopy results of (**a**). The experiment was repeated three times with similar results. Data are presented as mean ± SD. Statistical difference was evaluated using two-tailed Student's t-test. ns, $p > 0.05$.

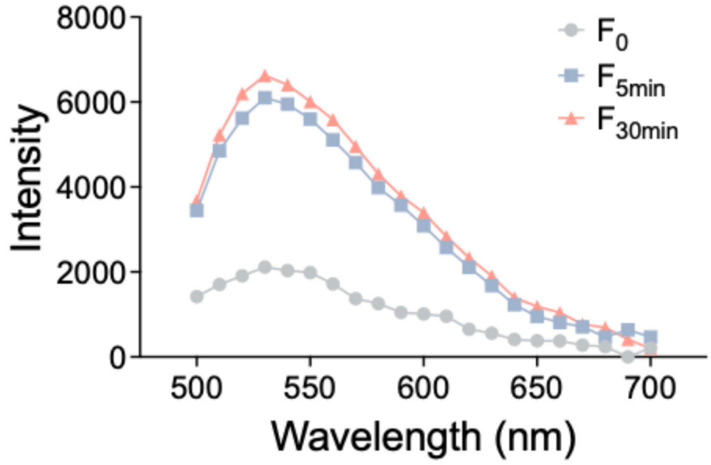

Fig. S3 Membrane fusion of N-7DHC-lipos. N-7DHC-lipos were labeled with hydrophobic NBD-PE and hydrophilic Texas-Red-PE, which form a FRET pair. The resulting nanoparticles were then incubated with H1299 cells. Samples were excited at 470 nm, and the emission spectra were recorded between 500 and 700 nm. The fluorescence of N-7DHC-lipos alone was recorded as F_0, while the fluorescence of N-7DHC-lipos after incubation with H1299 cells for 5 and 30 minutes were recorded as F_{5min} and F_{30min}, respectively. An increased fluorescence intensity at 530 nm indicates dye separation as a result of membrane fusion.

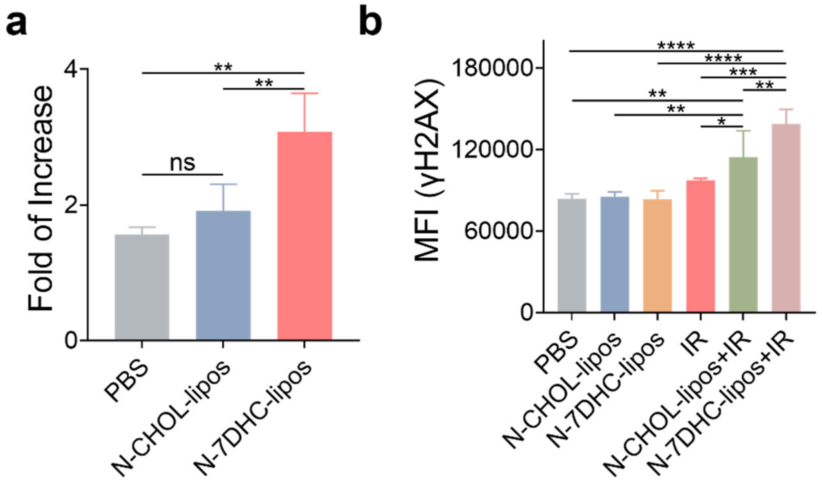

Fig. S4 Comparison between N-CHOL-lipos and N-7DHC-lipos in terms of inducing oxidative stress increase and DNA damage. H1299 cells were incubated with N-CHOL-lipos and N-7DHC-lipos (50 μg/mL) and then irradiated (5 Gy) (n=3 biologically independent samples). (**a**) Effect on the superoxide levels, measured by DHE. (**b**) Effect on DNA damage, measured by flow cytometry with anti-γH2AX-stained cells. Data are presented as mean ± SD. Statistical difference was evaluated using one-way ANOVA. *$p < 0.05$, **$p < 0.01$, ***$p < 0.001$, ****$p < 0.0001$; ns, $p > 0.05$.

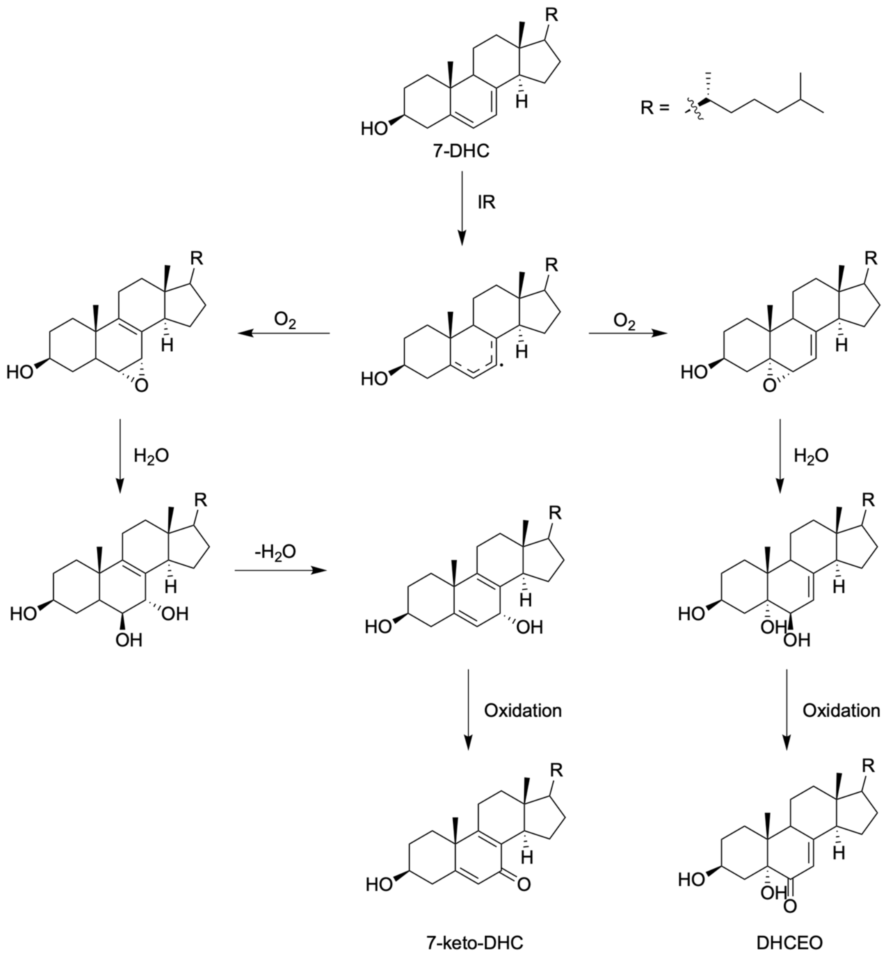

Fig. S5 Proposed mechanisms for 7DHC-induced autooxidation and the production of oxysterols under radiation.

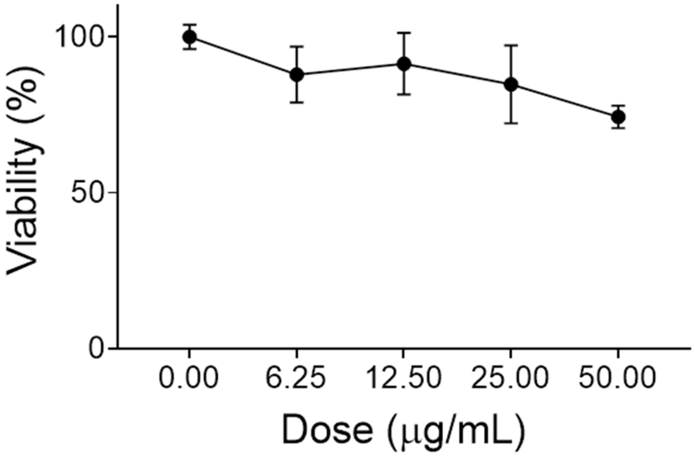

Fig. S6 Cell toxicity of N-7DHC-lipos, evaluated by the MTT assay at 48 hours of incubation (n=5 biologically independent samples). N-7DHC-lipos showed minimal toxicity when the concentration was below 50 μg/mL.

Test	Units	PBS		N-7DHC-lipos		Ref. Interval	
		Ave.	Std.	Ave.	Std.	Low	High
**WBC**	x 10^3/μl	3.83	1.10	4.70	1.18	0.8	10.6
**Neu#**	x 10^3/μl	0.98	0.23	1.00	0.38	0.23	3.60
**Lym#**	x 10^3/μl	2.65	0.85	3.52	0.72	0.60	8.90
**Mon#**	x 10^3/μl	0.12	0.03	0.12	0.06	0.04	1.40
**Eos#**	x 10^3/μl	0.07	0.02	0.05	0.00	0.00	0.51
**Bas#**	x 10^3/μl	0.01	0.01	0.00	0.01	0.00	0.12
**Neu%**	%	25.87	2.16	20.77	2.76	6.5	50.0
**Lym%**	%	68.67	2.66	75.40	3.04	40.0	92.0
**Mon%**	%	3.23	0.42	2.47	0.65	0.9	18.0
**Eos%**	%	1.90	0.00	1.27	0.25	0.0	7.5
**Bas%**	%	0.33	0.23	0.10	0.00	0.0	1.5
**RBC**	x 10^6/μl	9.42	0.52	9.21	0.39	6.50	11.50
**Hgb**	g/dl	14.53	0.91	14.10	0.36	11.0	16.5
**Hct**	%	43.23	2.47	42.63	1.57	35.0	55.0
**MCV**	fl	45.87	0.55	46.30	0.30	41.0	55.0
**MCH**	pg	15.43	0.50	15.30	0.26	13.0	18.0
**MCHC**	g/dl	33.57	0.68	33.00	0.35	30.0	36.0
**RDW%**	%	14.47	0.32	14.60	0.20	12.0	19.0
**Platelet**	x 10^3/μl	992.67	87.37	1075.33	102.16	400	1600
**MPV**	fl	5.40	0.10	5.33	0.12	4.0	6.2

Table S1. Summary of CBC results. Healthy BALB/C animals were administered i.v. N-7DHC-lipos (10 mg/kg) in PBS or PBS alone. Reported normal value ranges of indices are also listed. WBC, white blood cells; Neu, neutrophils, Lym, lymphocytes; Mon, monocytes; Eos, eosinophils; Bas, basophils; RBC, red blood cells; Hgb, hemoglobin; Hct, hematocrit; MCV, mean corpuscular volume; MCH, mean corpuscular hemoglobin; MCHC, mean corpuscular hemoglobin concentration; RDW, red cell distribution width; MPV, mean platelet volume.

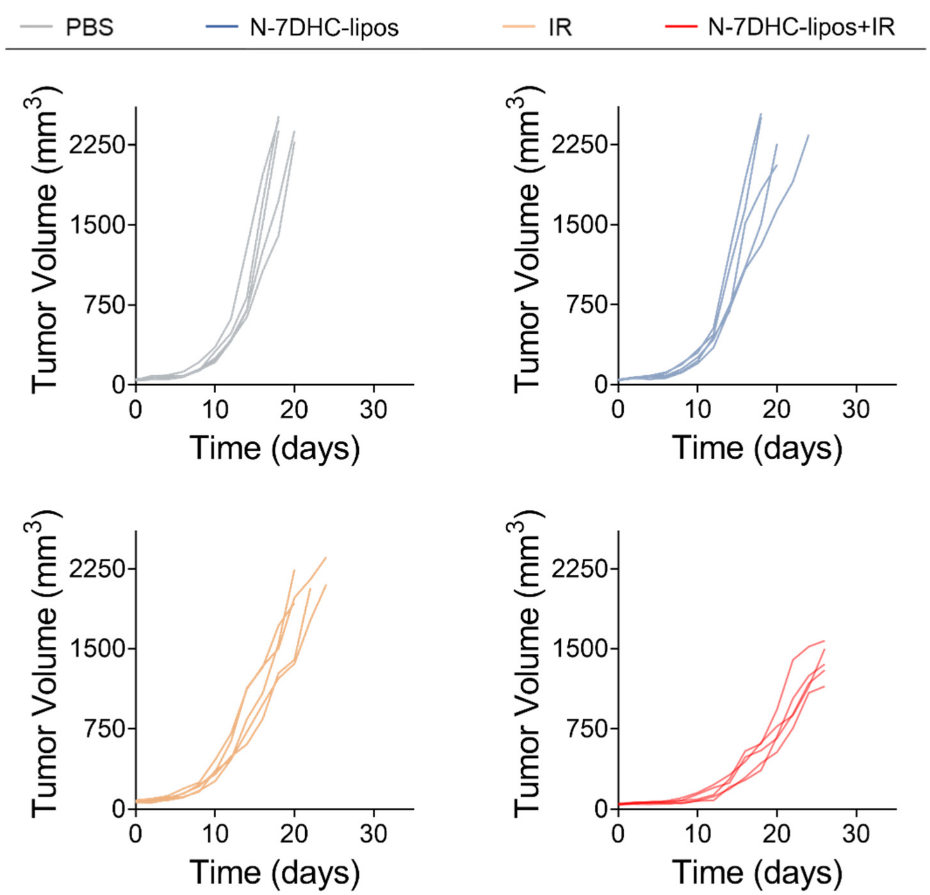

Fig. S7 Individual tumor growth curves. Study was performed in LLC1 tumor bearing C57BL/6 mice. N-7DHC-lipos (10 mg/kg) were injected i.v., followed by 5 Gy tumor irradiation at 24 hours. A total of three treatments were administered two days apart (n = 5 mice). For comparison, PBS alone, 7-DHC-lipos alone, and IR alone were tested.

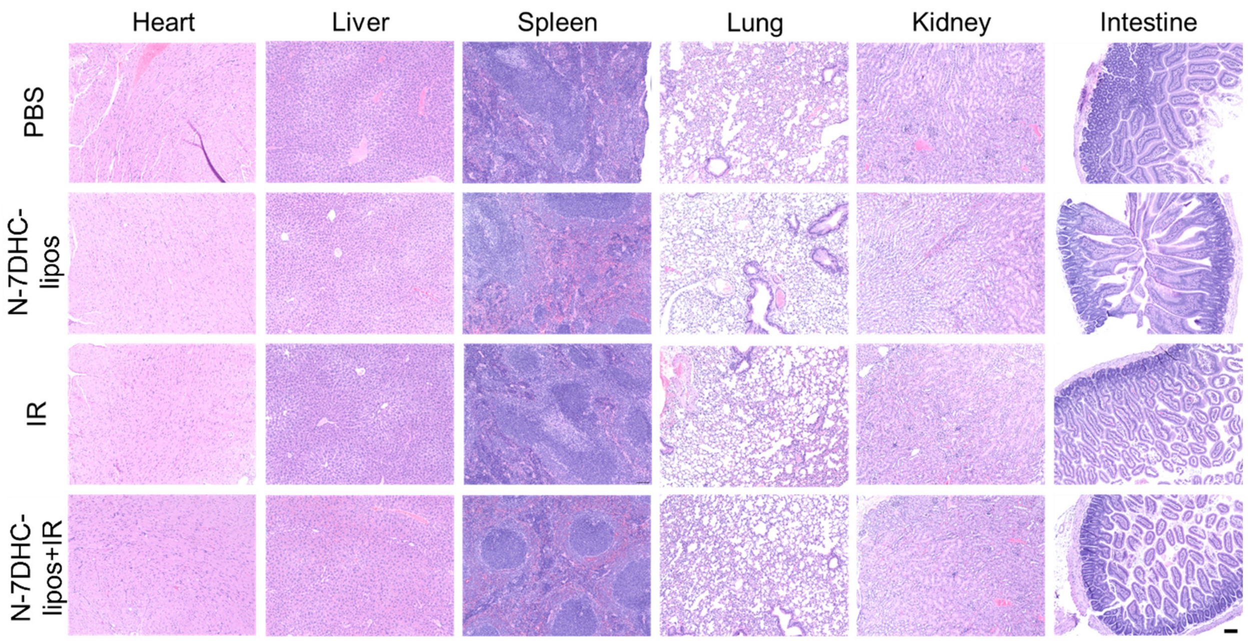

Fig S8 Post-mortem H&E staining of normal tissues, taken from H1299 tumor-bearing mice treated with PBS alone, N-7DHC-lipos alone, IR alone, and the combination of N-7DHC-lipos and IR. Scale bar, 200 µm.

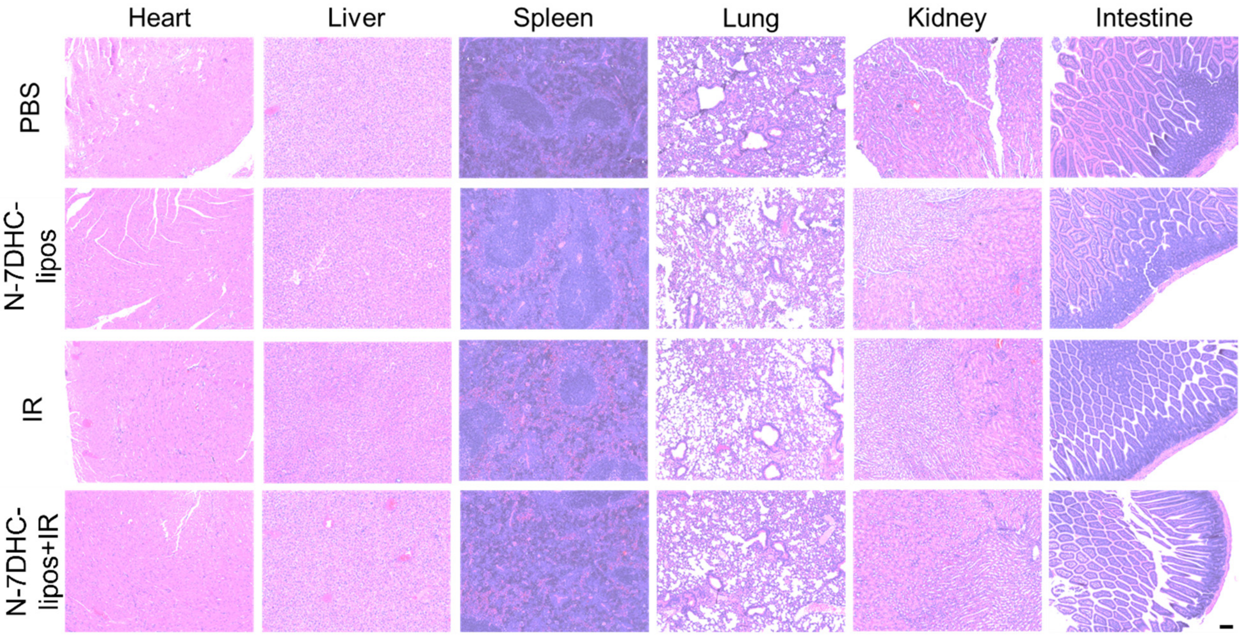

Fig. S9 Post-mortem H&E staining of normal tissues, taken from LLC1 tumor-bearing mice treated with PBS alone, N-7DHC-lipos alone, IR alone, and the combination of N-7DHC-lipos and IR. Scale bar, 200 μm.
